# Different Brain Network Connectomic Relationships Subserve Hand Dexterity During Task Versus Resting States in People with Brain Tumors

**DOI:** 10.1002/brb3.71032

**Published:** 2025-11-17

**Authors:** Timothy F. Boerger, Leon Taquet, Kaitlin Goetschel, Sarah Young, Jennifer Connelly, Jeffrey R. Binder, Brian D. Schmit, Max O. Krucoff

**Affiliations:** ^1^ Department of Neurosurgery Medical College of Wisconsin Milwaukee USA; ^2^ Institute For Health and Equity Medical College of Wisconsin Milwaukee USA; ^3^ Department of Neurology Medical College of Wisconsin Milwaukee USA; ^4^ Department of Biomedical Engineering Marquette University and Medical College of Wisconsin Milwaukee USA; ^5^ Department of Biophysics Medical College of Wisconsin Milwaukee USA

## Abstract

**Purpose:**

The purpose of this study was to explore different brain network connectomic relationships subserving hand dexterity in patients with contrast‐enhancing brain tumors during task and resting states.

**Method:**

We measured hand dexterity, resting state functional connectivity, and task‐based functional connectivity in 21 participants with newly diagnosed brain tumors. Hand dexterity was measured using the 9‐hole peg test (9HPT), and patient‐reported outcomes were assessed with the Duroz Hand Index (DHI).

**Findings:**

We discovered the following: (1) The inability to complete dexterous tasks is most associated with *low* somatomotor‐basal ganglia network connectivity during rest but *high* somatomotor‐basal ganglia network connectivity during tasks; (2) in the subgroup of better dexterous performers, resting somatomotor‐salience connectivity is *higher* in people with *poor* dexterity—a relationship that holds true in healthy adult subjects from the Human Connectome Project (HCP), suggesting it has fundamental importance; and (3) connectomic measurements were stronger predictors of dexterous performance than classical variables of tumor (i.e., size, grade, or anatomical location).

**Conclusions:**

These data suggest that connectomic correlates of dexterity are different in resting and task‐based states. Additionally, our data suggest a threshold level of somatomotor‐to‐basal ganglia connectivity is required to accomplish dexterous movements, and, in the cases of appropriately preserved somatomotor‐to‐basal ganglia connectivity, salience‐somatomotor connectivity then becomes the dominant connection facilitating performance in a hierarchical fashion. These findings have fundamental implications for both surgical planning and neuromodulation‐based rehabilitation.

## Graphical abstract

Key findings of this study include that poor dexterity in patients with brain tumors is related to reduced somatomotor connectivity to the basal ganglia and increased connectivity with the salience networks. Lesions in the anterior hand knob are most associated with such reduced dexterity and connectivity.

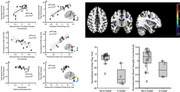

## 1 Introduction

Hand dexterity is a physiologically complex function that is critical to everyday life. (Amidei and Kushner 2015; Řasová et al. 2024) From a neurological systems perspective, its execution requires integrating motor, sensory, planning, visual, attention, and error correction functions, as well as bidirectional and plastic communication between the brain, spinal cord, peripheral nerves, and musculoskeletal systems. (Rinne et al. 2018; Kandel et al. 2012; Sobinov, and Bensmaia 2021) While it is well‐known that brain lesions can impact upper extremity function, lesion location and volume often only partially explain the level of observed dysfunction in cases of higher‐order deficits, such as dexterity. (Jingshan et al. 2019; Lim et al. 2020) To bridge this gap in knowledge, a variety of connectomic predictors of poor dexterity have been identified across various neurological pathologies. For example, in stroke, dexterity has been related to resting‐state intrinsic connectivity of the somatomotor and salience networks. (Rinne et al. 2018) In multiple sclerosis, dexterity has been associated with resting‐state seed‐to‐voxel‐based connectivity of the basal ganglia to the supplementary motor area. (Romanò et al. 2024) In people with brain tumors, dexterity has been associated with connectivity between the primary motor cortex and the supplementary motor area and salience networks. (Otten et al. 2012; Newbold et al. 2021; Gordon et al. 2023) Thus, it is possible that brain tumors may affect dexterity through under‐characterized changes in somatomotor connectivity to other brain networks.

Our group is particularly interested in studying brain tumors because (1) surgery and radiation play a large role in their treatment, (2) guidance for such interventions could be improved with better knowledge of anatomical relationships subserving dexterity, and (3) dexterity plays a large role in quality of life and Karnofsky Performance Score (KPS), both of which directly impact survival. (McGirt et al. 2009; Rahman et al. 2017) Therefore, here we designed a prospective study to test whether a data‐driven framework of connectivity between the ipsilesional somatomotor network and the executive control network, salience network, contralesional cerebellum, basal ganglia, thalamus, and/or secondary somatomotor or contralesional somatomotor networks would predict dexterity (Analysis 1). We additionally assessed if connectivity relationships from Analysis 1 might be different in resting and task states (Exploratory Analysis 1). We then examined connectomic predictors of dexterity in the subset of better‐performing participants able to complete the dexterity test within certain parameters (Exploratory Analysis 2). Next, we conducted a replication analysis on publicly available data from participants in the Human Connectome Project (HCP) to determine if our identified main predictor from Exploratory Analysis 2 generalized to healthy adults (Exploratory Analysis 3). Additionally, we conducted an exploratory analysis of predictors of self‐reported hand function to determine if objective and self‐reported dexterity correlated with the same primary variable (Exploratory Analysis 4). Finally, we analyzed conventional tumor lesion and anatomy metrics (e.g., volume, corticospinal overlap, etc.) (Exploratory Analysis 5) and conducted a voxelwise lesion symptom mapping analysis of dexterity (Exploratory Analysis 6).

## 2 Methods

### 2.1 Brain Cancer Cohort

For this prospective, observational study, we recruited 23 people with singular, newly diagnosed, contrast‐enhancing mass lesions with clinical indications for surgical resection (Supplementary Materials S1). Participants were excluded if they had previous brain surgery or a history of other potentially confounding brain or spinal cord injury or disease. All participants underwent functional magnetic resonance imaging (fMRI) for surgical planning. One participant was excluded because tissue pathology confirmed that the disease process was an ischemic stroke, leaving 22 participants for analysis after surgery. This study was approved by the Institutional Review Board at the Medical College of Wisconsin (protocol # PRO00042089) and conformed to the Declaration of Helsinki. Informed consent was obtained from all participants. Of the 22 participants who completed the study, 12 were male. Fifteen participants were diagnosed with a high‐grade glioma, 5 with a brain metastasis, and 2 with radiation necrosis secondary to radiation treatments for lung cancer. Four patients had preoperative recurrent seizures. Participant ages (Table 1) were 58.68 ± 10.95 (range 39–76) years old.

**TABLE 1:** Clinical data of respective participants.

ID	Age	Sex	Dom hand	Lesioned hemisphere	Cancer type	Primary (if Met)	Lesion lobe	MGMT Meth.	IDH WT	Lesion volume (cm^3^)	MMT (CL Hand)	MMT (IL Hand)
1	63	M	R	L	Met	NSCLC	F			14.03	5	5
2	39	M	R	L	GBM		I	Y	Y	31.71	4.5	5
3	76	F	R	R	GBM		P	N	Y	95.17	4.5	5
4	67	F	R	L	GBM		F	Y	Y	16.06	4.5	5
5	53	M	R	R	GBM		F	N	Y	14.74	4.5	5
6	61	M	R	R	GBM		F	Y	Y	7.18	4.5	5
7	67	F	L	R	GBM		F	Y	Y	20.57	5	5
8	58	M	L	R	Met	RCC	F			7.73	4	5
9	70	M	R	L	Met / RN	Uro	F			23.27	5	5
10	49	M	L	L	RN	NSCLC	F/P			23.62	5	5
11	57	F	L	L	RN	NSCLC	F/P			1.50	5	5
12	62	F	L	L	GBM		F	Y	Y	62.14	4.5	5
13	57	M	L	R	GBM		T	N	Y	27.40	5	5
14	49	F	R	L	GBM		F	Y	Y	24.46	5	5
15	51	M	L	L	GBM		P	N	Y	34.08	5	5
16	70	M	R	L	Met	NSCLC	P			12.08	5	5
17	57	F	R	R	GBM		F	Y	Y	65.14	4.5	5
18	72	F	R	L	GBM		F	N	Y	11.05	5	5
19	66	F	R	R	GBM		P	Y	Y	48.47	5	5
20	39	M	R	L	GBM		P	N	Y	9.92	5	5
21	69	F	R	L	GBM		P	N	Y	17.72	5	5
22	39	M	L	R	Met	Esoph	F			29.66	3	5

*Note*: Participant 10 was withdrawn from the study as pathology determined the lesion was an ischemic stroke.

Abbreviations: Esoph = Esophageal adenocarcinoma, F = female, F = frontal, GBM = glioblastoma multiforme, I = insular, ID = identifier, L = left, M = male, Met = metastasis, MMT = manual muscle test, N = no, NSCLC = non‐small cell lung cancer, P = parietal, R = right, RCC = renal cell carcinoma, RN = radionecrosis, t = temporal, Uro = urothelial carcinoma, Y = yes.

### 2.2 Imaging Protocol

Participants underwent preoperative, clinically indicated MRIs, including 3D T1 with and without contrast, T2 fluid attenuated inversion recovery (FLAIR), and standard T2 imaging. These images were used for automated tumor segmentation. (Pati et al. 2020) Resting state (rs) fMRI was also obtained on all participants (duration = 7–10 min) TR = 2‐3.157 s, TE = 0.03 s, matrix = 64 × 64 − 80 × 80, slice thickness 3–5 mm, flip angle = 90). Scan parameters were based on clinical protocol, and differences between participants were accounted for through statistical regression. Additionally, 20 participants underwent clinically indicated task‐based (TB) fMRI for motor and/or language mapping. The task for locating hand motor involved sequentially tapping from the index finger through to the small finger and then to the thumb in a 20 s active / 20 s resting block design.

### 2.3 fMRI Processing

fMRI data were processed using bias field correction, motion correction, spatial smoothing (6 mm full width half maximum), independent component analysis‐based denoising (ICA‐AROMA) (Pruim et al. 2015; Pruim et al. 2015), registration to standard space, detrending, scrubbing with trilinear interpolation using DVARS (D = “derivative,” VARS = root mean square variance of BOLD signal), (Power et al. 2012), and bandpass filtering (0.01 < *ƒ* < 0.08 Hz) to remove persistent noise. Previous work has demonstrated that ICA‐AROMA and scrubbing (e.g., DVARS) are as effective at removing noise as expanded physiological (e.g. aCompCor) and motion regressors (e.g., the Friston 24‐parameter model); however, combining all four of these processes risks overly reducing temporal degrees of freedom. (Parkes et al. 2018; Grajauskas et al. 2019). Therefore, ICA‐AROMA and motion scrubbing were used in the current study. Note that global signal regression was not used in this study. While removing global signals improves brain‐behavior relationships, it can introduce spurious anti‐correlations. (Li et al. 2019; Murphy and Fox 2017) However, partial correlation was used for calculating functional connectivity (see below), which accounts for global signal while potentially introducing less artefactual anticorrelations. (Murphy and Fox 2017) When gross anatomical distortions remained after initial registration, we serially re‐registered images to standard space (Supplementary Materials S2). Timeseries data were extracted using a 17‐network cortical atlas representing subdivisions of the networks described above and supplemented with subcortical areas for network analyses (Yeo et al., 2011) For parcel‐level analyses, a 400‐parcel atlas was used. (Schaefer et al. 1991) For task‐based connectivity, we regressed the gamma‐convolved block design from the time series. Functional connectivity of the residual BOLD signal following denoising and model regression for the task‐based connectivity analysis was calculated as the partial correlation coefficient and converted to z‐scores. Following denoising, quality control was conducted on the functional connectivity analyses. Specifically, framewise displacement calculated prior to any denoising steps was correlated with post‐processed connectivity to determine if our connectivity findings were potentially related to residual noise. (Power et al. 2012; Parkes et al. 2018)

### 2.4 Hand Dexterity Testing

Motor testing consisted of the 9‐hole peg test (9HPT) and Duruoz hand index (DHI), which were collected on all participants. The 9HPT is a well‐established measure of hand dexterity where participants sequentially pick up and place 9 pegs into 9 holes with each hand (Ultrassist, Dongguan, Guangdong, China) as fast as possible, and time to completion for each hand is the score (maximal time allowed is 120 s). (Pernet et al. 2016; Beebe, and Lang 2009) Participants performed a single practice trial and a single test trial consistent with NIH Toolbox Protocols. (Reuben et al. 2013) Time to completion was normalized to published age, sex, and hand‐stratified normative data.

The DHI (Duruöz et al. 1996) is an 18‐item questionnaire for self‐assessment of independence in activities of daily living. It is scored on a 6‐point Likert scale ranging from 0 (can perform task without difficulty) to 5 (unable to perform), resulting in a score from 0 to 90, where increasing values represent greater dysfunction. (Duruöz et al. 1996)

### 2.5 HCP Cohort

All participants from the full HCP dataset (Van Essen et al. 2013) (1200) were analyzed to identify participants below the 10^th^ (low, *n* = 100) and above 90^th^ (high, *n* = 100) percentiles for hand dexterity (measured with the 9HPT). Of these 200 participants, we randomly selected 21 each from the “high” and the “low” dexterity groups for further analysis of rs fMRI data. For this analysis, we specifically used the ICA‐FIX denoised rs fMRI dataset. Details on these processing steps are provided elsewhere (Van Essen et al. 2013), and greater details for this study are provided in the Supplementary Materials. All participants from the HCP were < 36 years old. The low dexterity group was slightly older (26.86 ± 3.02 years vs. 24.67 ± 6.57, 95% CI = 0.11–4.27 years, *p* = 0.04) and more frequently male (12 male vs. 6 male, odds ratio 0.30, 95% CI = 0.08–1.08, *p* = 0.11) than the high dexterity group.

### 2.6 Lesion‐Symptom Mapping Analysis

A lesion‐symptom mapping analysis was conducted using the support vector regression lesion‐symptom mapping (SVR‐LSM) toolbox. (DeMarco and Turkeltaub 2018) The SVR‐LSM toolbox is an open‐source MATLAB implementation of multivariate lesion‐symptom mapping and is run through MATLAB. The SVR‐LSM analysis corrects for the effects of lesion volume via regressing out the effects of tumor volume from both lesion masks and dexterity data. (DeMarco and Turkeltaub 2018) A threshold of three participants (van Grinsven et al. 2023) having a tumor in a given voxel was required for inclusion. The default hyperparameters for the SVR‐LSM toolbox (Cost/Box Constraint = 30.00, Sigma = 0.45, Epsilon = 0.10) were used for this study.

### 2.7 Statistical Analyses

Statistical analyses were performed in MATLAB v2018a on a 64‐core computing cluster. All tests were two‐tailed. Outliers were detected with *isoutlier* set to identify outliers based on a threshold of 1.5× interquartile range. For our primary analysis, 1 outlier was identified (analyzed *n* = 21). Data for this individual was included in other analyses in which he was not an outlier. Our primary analysis was the relationship between connectivity and dexterity across the entire brain tumor cohort. We performed a stepwise regression (*stepwiselm*) with the *p*‐value for entry set to 0.05/11 (e.g., Bonferronni adjustment) (i.e., 0.0045). Subsequent analyses were done with an alpha (*α*) of 0.05. Stepwise regression was also used for several secondary analyses *(Exploratory Analyses 1, 2, and 4)*. Likewise, for *Exploratory Analysis 3*, we used a logistic regression (*stepwiseglm*) with alpha set to 0.05. For these regressions, 95% confidence intervals or regression coefficients were obtained using *coefCI*. A permutation test with 10,000 permutations with false discovery rate correction was used for parcel‐level data for *Exploratory Analysis 3*. Wilcoxon Signed Rank tests were performed to determine if the contralesional hand was different from the ipsilesional hand with and without data from individuals who could not complete the dexterity test. The SVR‐LSM analysis was permuted 10,000 times, and a voxelwise corrected alpha was set to 0.005, and a cluster correction was set to *p* < 0.05. (DeMarco and Turkeltaub 2018) Further, to demonstrate model stability across statistical frameworks, we employed a least absolute shrinkage and selection operator (e.g., LASSO) regression model with leave‐one‐out cross‐validation (LOOCV) and bootstrapping by 1,000,000 replications in R (v4.4.3) using the following libraries: *HDCI*, *doParrallel*, and *car*. Bootstrapped 95% confidence intervals of LASSO beta coefficients that did not cross 0 were considered statistically significant features. All final multilinear regression models were cross‐validated with LOOCV to determine the predictive *R* ^2^ of the final model. *R* ^2^ coefficients were then bootstrapped to obtain 95% confidence intervals.

## 3 Results

9HPT performance was lower in contralesional hands, including (*p* = 0.00012) and excluding (*p* = 0.0013) participants unable to complete the test. One individual's motor testing and functional connectivity data were consistently outside the 1.5× interquartile range (ID 23), so they were considered an outlier and excluded from this analysis.

### 3.1 Analysis 1. Resting‐State Prediction of Dexterity in all Brain Tumor Participants

**FIGURE 1:**
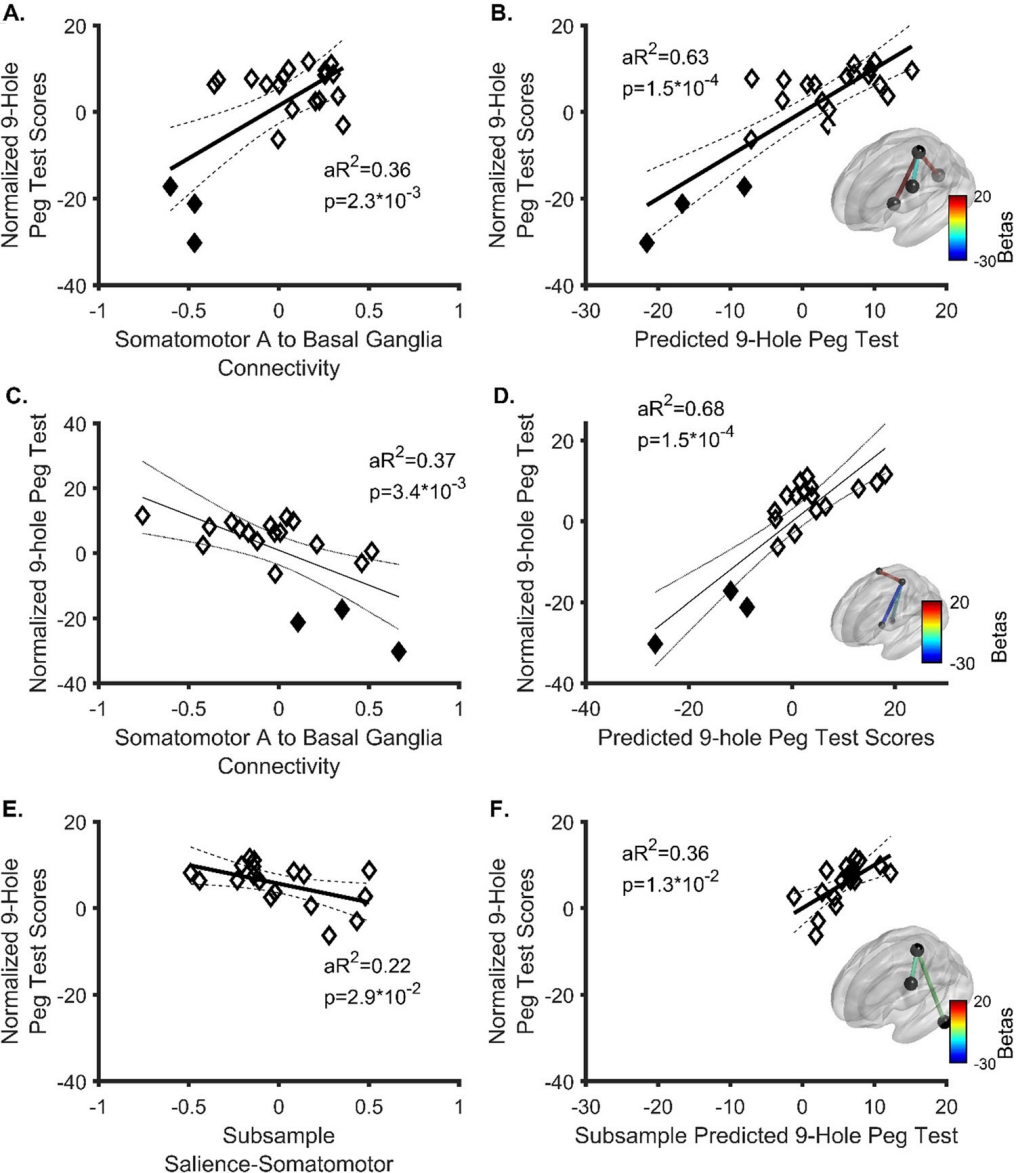
Results of stepwise regression predicting dexterity performance from functional connectivity in patients with brain cancer. Filled diamonds represent the scores of patients unable to complete the 9‐hole peg test in < 120 s who were subsequently excluded from subanalyses. **(A)** The strongest predictor of dexterity performance in the full sample was connectivity between the somatomotor A and the basal ganglia networks. One outlier was removed (*n* = 21). The bootstrap 95% CI for the *R* ^2^ statistic was 0.09 – 0.82, **(B)** Results of stepwise regression (*n* = 21) run to natural termination to predict dexterity. Significant predictors included Somatomotor A connectivity to the basal ganglia, salience A, and contralesional visual central networks 95% CI for the *R* ^2^ statistic was 0.23–0.91, (C) The strongest task‐based connectomic predictor of dexterity was connectivity between the somatomotor A and basal ganglia networks. Note especially the reversal of the slope for Somatomotor A to Basal Ganglia relative to panel A, **(D)** Results from a stepwise regression run to natural termination. Significant predictors included somatomotor A connectivity to the basal ganglia, contralesional dorsal attention A, and contralesional default mode C networks. The inset shows the applicable connectome edges’ coefficients (betas) for the stepwise regression with dexterity, **(E)** The strongest predictor of dexterity in the subset of patients able to complete the 9‐hole peg test (*n* = 18) was connectivity between the somatomotor A and salience A networks, and **(F)** The results of the stepwise regression run to natural termination. Significant predictors included somatomotor A connectivity to the salience A and ipsilesional cerebellum. aR2 = adjusted *R* ^2^. Considering the respective models for A‐B (including participants unable to perform dexterous tasks as well as those able to perform the dexterous task) versus E‐F (only those able to complete dexterous tasks), it is evident that somatomotor‐to‐basal ganglia connectivity is most important when considering both subsets (able and unable to complete the dexterous task). Therefore, we propose there may be a threshold such that, when a lesion induces at least moderate anticorrelation of somatomotor‐to‐basal ganglia connectivity at rest (panel A), individuals will be unable to complete the dexterous task, which is otherwise hierarchically correlated primarily with by somatomotor‐to‐salience connectivity.

With strict entry criteria (*p* < 0.0045), the strongest predictor of dexterity was the ipsilesional somatomotor‐to‐basal ganglia network (Figure 1, *F*_2, 19_ = 12.68, *β* = 24.39 (95% CI = 10.06–38.73), *p* = 0.002). Notably, this relationship was positive, and there was an apparent threshold effect (Figure 1A). Follow up exploratory analyses with the entry *p*‐value loosened to 0.05 and expanded to all networks (*F*_4, 17_ = 13.1, *p* = 0.0001) added the ipsilesional salience (*β* = −10.65 (95% CI = −19.85 to −1.44), *p* = 0.026) and the somatomotor‐to‐contralesional visual network (*β* = 11.79 (95% CI = 3.82–19.77), *p* = 0.006). A full model cross‐validated with LOOCV including somatomotor connectivity to the basal ganglia, salience, and visual networks predicted 40% (Bootstrap 95% CI = 6%–73%) of the variance in hand dexterity. Partial regressions of the final model are demonstrated in Supplementary Materials S3. Regression results for the full sample, including the outlier individual, are presented in Supplementary Materials S4. With the outlier included, only the somatomotor‐to‐basal ganglia connectivity was associated with dexterity (*β* = 25.98 (95% CI = 8.04–43.91), *p* = 0.0067). Follow up linear regression accounting for variability of pathology (glioblastoma (GBM) vs. non‐GBM) remained significant (*F*_5, 16_ = 9.50, aR^2^ = 0.63, *p* = 0.004) with somatomotor‐to‐basal ganglia (*β* = 28.44 (95% CI = 16.73–40.14), *p* = 0.0001) and somatomotor‐to‐salience (*β* = −11.07 (95% CI = ‐20.69 to −1.45), *p* = 0.027) and somatomotor‐to‐contralesional visual central (*β* = 11.11 (95%CI = 2.44–19.78), *p* = 0.015) (Figure 1B) remaining as significant, independent predictors of dexterity. Pathology type (*β* = 1.86 (95%CI = −5.86 to −9.59), *p* = 0.62) was not a significant, independent predictors of dexterity. Therefore, our data suggest that the strongest predictor of hand dexterity was basal ganglia‐to‐somatomotor network connectivity, followed by salience‐to‐somatomotor network and visual central‐to‐somatomotor network. Moreover, based on rs fMRI connectivity, somatomotor‐to‐basal ganglia connectivity was positively related to dexterity.

### 3.2 Exploratory Analysis 1. Task‐Based Functional Connectivity Prediction of Dexterity in Study Participants

Twenty participants also completed a finger‐tapping task‐based fMRI as part of their preoperative workup. In this group, somatomotor‐to‐basal ganglia connectivity inversely predicted dexterity (*β* = −21.55 (95% CI = −34.93 to 8.16), *p* = 0.003) during the task. Additional regression model steps (Figure 1C; *F*_4, 15_ = 13.6, aR^2^ = 0.68, *p* = 0.0002) resulted in inclusion of the contralesional dorsal attention network (*β* = 11.94 (95% CI = 4.33–19.54), *p* = 0.004) and contralesional default mode network (*β* = −12.45, *p* = 0.011). Altogether, these connections, in a LOOCV analysis, predicted 53.3% (bootstrap 95% CI = 17.3%–78.7%) of the variance in dexterity. Therefore, as with resting state connectivity, finger tapping task‐based connectivity between the somatomotor and basal ganglia network was the strongest predictor of dexterity without (Figure 1D, Supplementary Materials S5, and with (*β* = −23.68 (95% CI = −39.49 to 7.86), *p* = 0.006), Supplementary Materials S5) the outlier included. Notably, the results from Analysis 1 and Exploratory Analysis 1 together indicate that the relationship between somatomotor‐to‐basal ganglia connectivity and dexterity inverts based on rest versus task state data.

### 3.3 Exploratory Analysis 2. Subanalysis of Better‐Performing Participants Able to Complete Dexterity Test Within 2 Min

Subsequently, we performed an exploratory analysis of the subset of patients able to complete the task in less than 120 s (*n* = 18). Somatomotor network connectivity to the salience/ventral attention network (*β* = −7.46 (95% CI = −14.45 to 0.48), *p* = 0.038) and ipsilesional cerebellum (*β* = −5.00 (95% CI = −9.97 to 0.04), *p* = 0.049, Supplementary Materials S6) explained 36% of the variance in hand dexterity. The strongest parcel‐level predictor of dexterity in this group was a parcel within the salience network near the operculum (Supplementary Materials S7, *β* = −45.14, aR^2^ = 0.56, *p* = 0.00034). Therefore, somatomotor‐to‐salience connectivity was *negatively* associated with dexterous ability at rest, and this relationship was most strongly derived from peri‐opercular structures.

### 3.4 Exploratory Analysis 3. Prediction of Dexterity in Healthy Controls

**FIGURE 2:**
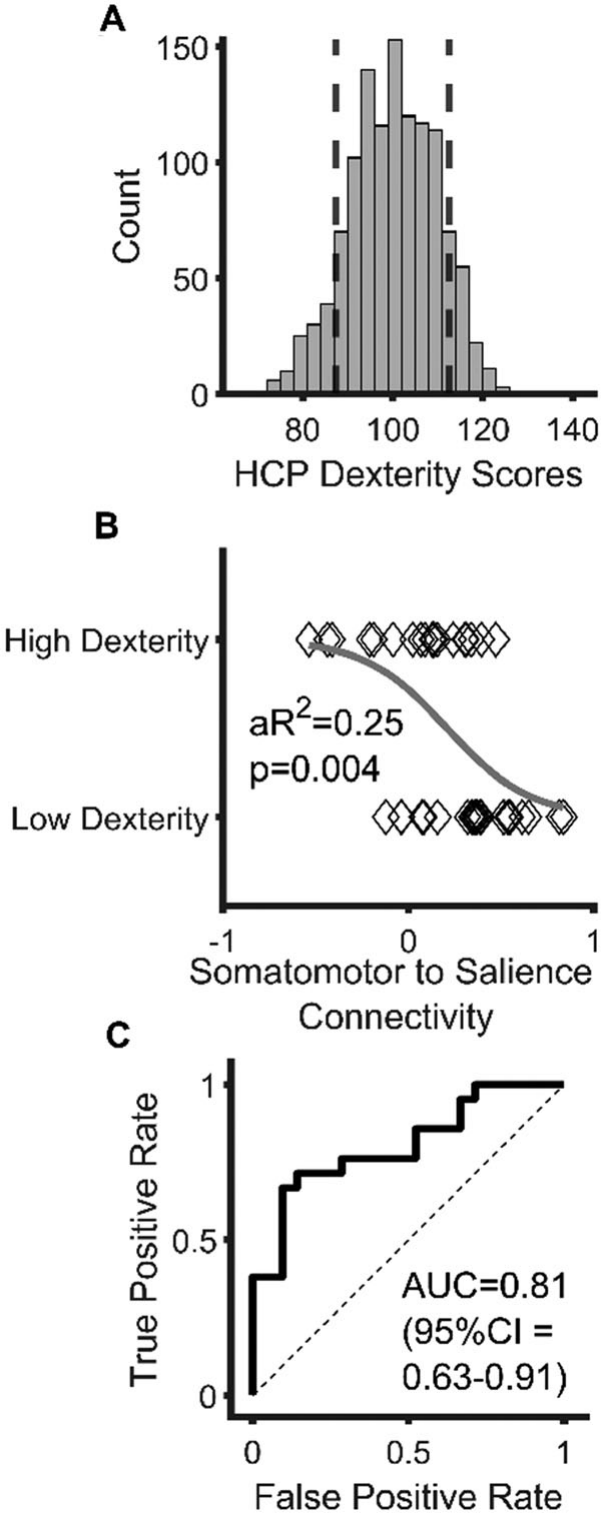
Results of Human Connectome Project analysis predicting dexterity in > 90^th^ versus < 10^th^ percentiles. **(A)** Distribution of dexterity scores in HCP data. Scores are normalized to a whole population mean of 100 and standard deviation of 15 per the scoring protocol of the NIH Toolbox. Vertical dashed lines represent the boundary of the 10^th^ and 90^th^ percentiles of hand dexterity scores, **(B)** Scatter plot with probability curve of somatomotor‐to‐salience A connectivity from the HCP versus dexterity, and **(C)** Receiver operator characteristic curve for the prediction of the decile categories. Abbreviations: aR^2^ = adjusted *R* ^2^, AUC = area under the curve, HCP = Human Connectome Project.

To validate our results from Exploratory Analysis 2, we performed a similar stepwise logistic regression to compare the difference between high dexterity (> 90^th^ percentile) and low dexterity (< 10^th^ percentile) participants in the human connectome project (Figure 2A). Consistent with our findings in brain tumor patients who could complete the 9HPT, somatomotor‐to‐salience/ventral attention connectivity significantly predicted high versus low hand dexterity in HCP participants (Figure 2B. *β* = ‐3.50, Exp(*β*) = −0.03, *p* = 0.007). A receiver operator characteristic (ROC) curve was significant (Figure 2C, AUC = 0.81 (95% CI = 0.63–0.91), *p* = 0.002). No other between‐network connections were significant predictors of dexterity (p>0.05). After statistically controlling for age and sex differences on dexterity and connectivity, somatomotor‐to‐salience remained significantly related to dexterity (*F*_2, 40_ = 12.00, aR^2^ = 0.21, *p* = 0.001).

As a sub‐analysis at the parcel‐to‐parcel level, the strongest predictive salience network parcel was the left perioperculum (Hedge's *g* = ‐1.19, *p*_FDR_ = 0.029, Supplementary Materials S8). Anatomically, this parcel straddles the sulcus between the pars opercularis of the inferior frontal gyrus and the pre‐central gyrus. Therefore, in healthy adults, dexterous performance is negatively related to connectivity between the salience and somatomotor networks, with the strongest correlation between the somatomotor and perioperculum.

### 3.5 Exploratory Analysis 4. Connectomic Predictors of Self‐Reported Hand Function in all Study Participants

Connectomic predictors of self‐reported hand function (DHI) were similar to 9HPT performances. Using a stepwise regression, somatomotor‐to‐basal ganglia network connectivity (*β* = ‐65.09 (95% CI = −93.80 to 36.38), aR^2^ = 0.52, *F*_2, 19_ = 22.52, *p* = 0.00014, Supplementary Materials S9) was the strongest imaging predictor of DHI. The next strongest predictor of dexterity based on a stepwise regression (*F*_3, 18_ = 16.3, *p* = 0.00009) was connectivity between the somatomotor A network with the contralesional somatomotor B network (*β* = −24 (95% CI = −46.12 to 1.89), (Supplementary Materials S8). DHI and 9HPT scores were strongly inter‐related (Supplementary Materials S8, *F*_2, 20_ = 67.79, aR^2^ = 0.76, *β* = ‐1.92 (95% CI = −2.41 to 1.44), *p* = 0.000000075).

### 3.6 Exploratory Analysis 5. Conventional Tumor Metrics Poorly Predict Dexterity

Analysis of lesion volume and volume of lesion overlap with the corticospinal tract, somatomotor network, or hand knob gray matter specifically as predictors of dexterity were all non‐significant (Supplementary Materials S10; *R* ^2^ < 0.14, p>0.05). Additional statistics are reported in the supplement.

### 3.7 Exploratory Analysis 6: Voxelwise Lesion Symptom Mapping Analysis of Poor Dexterity

**FIGURE 3:**
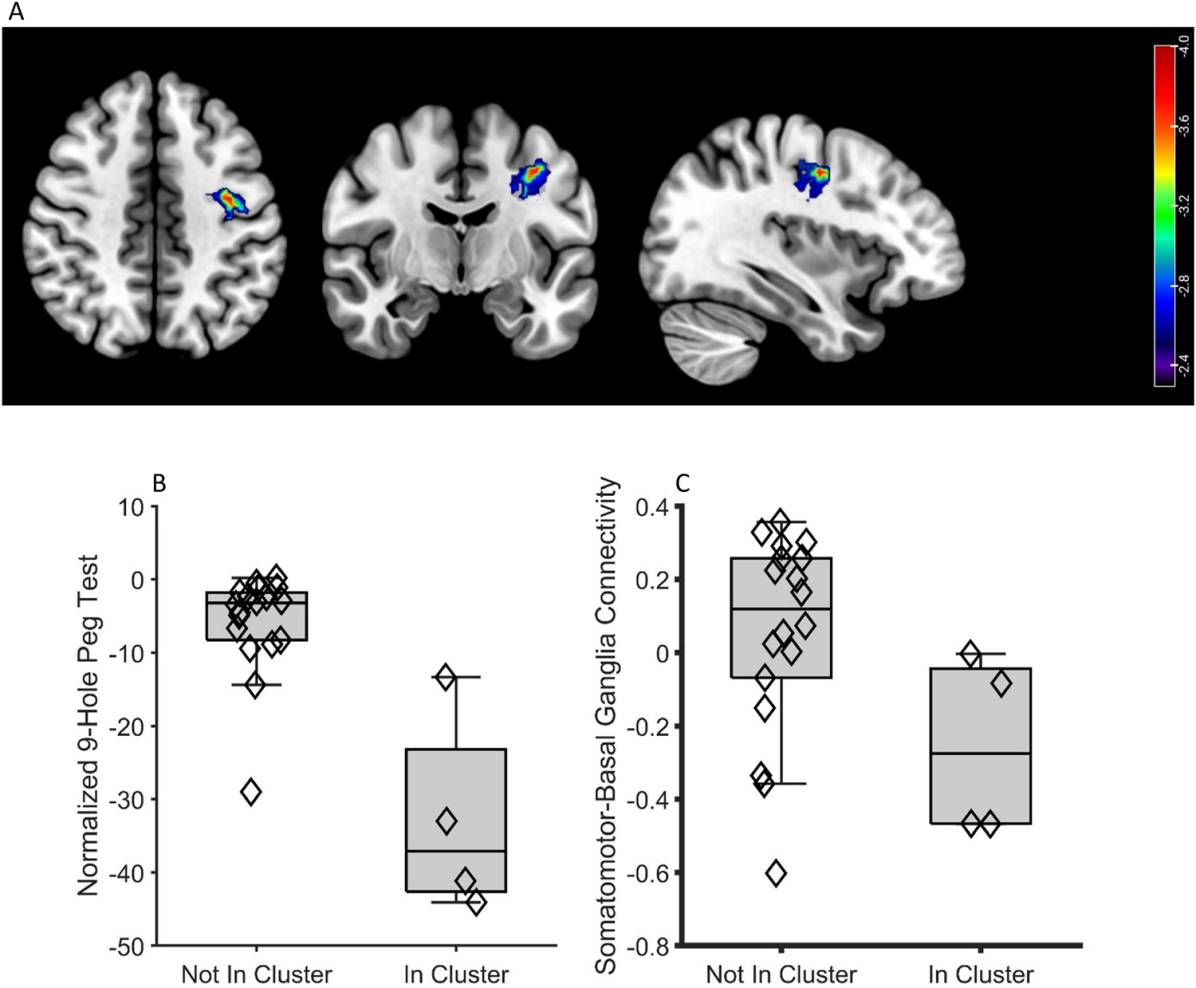
Patients with tumors in the inferior gyral white matter of the precentral gyrus have disrupted dexterity and somatomotor‐to‐basal ganglia connectivity. **(A)** Topography of lesion location causing reductions in hand dexterity, **(B)** Distributions of dexterity scores depending on whether a given patient has a lesion within the most significant cluster, and **(C)** Distribution of somatomotor‐to‐basal ganglia connectivity depending on if a patient had a tumor overlapping the most significant cluster.

Support vector regression‐based lesion symptom mapping (*n* = 22) demonstrated that involvement of a lesion in the inferior gyral white matter of the hand knob most strongly and significantly predicted poor dexterity (Figure 3A, *Z* = −3.72, permutation test *p*_FDR_ < 0.005). Presence of a tumor within this area accounted for 62% of the variance in dexterity (*F*_2, 20_ = 34.6, *β* = ‐27.09 (95% CI = −36.69 to 17.48), *p* = 0.000009). Additionally, tumor presence in this region significantly predicted worse connectivity (Figure 3B, *R^2^* = 0.14, *p* = 0.047) between the somatomotor and basal ganglia networks (Figure 3C), implying that this region may contain a critical connection between them.

### 3.8 Quality Control of Functional Connectivity

To assess quality control, we correlated pre‐processed framewise displacement with post‐processed connectivity. The only connectivity edges that were associated with pre‐processed framewise displacement were the ipsilesional somatomotor network connectivity to the contralesional thalamus (*r* = −0.47, *p* < 0.05) and the contralesional limbic B network representing the orbitofrontal cortex (*r* = −0.44, *p* < 0.05). Ipsilesional somatomotor connectivity to the ipsilesional basal ganglia (*r* = 0.06, *p* = 0.80) and salience networks (*r* = 0.0024, *p* = 0.99) was not strongly or significantly associated with pre‐processed framewise displacement.

### 3.9 LASSO Feature Selection With Bootstrapping

We additionally applied a two‐stage prediction algorithm based on LASSO feature selection with bootstrapping and (LOOCV) followed by OLS regression based on the LASSO features to further validate our predictions. Statistical results for these analyses are included in the supplement (Supplementary Materials S11–S16). These supplemental figures present the bootstrap 95% confidence intervals of LASSO features as well as the cross‐validated predicted versus observed scatter plots for the OLS regression models along with reporting bootstrapped 95% confidence intervals for the OLS regression *R* ^2^. Our resting state correlates of dexterity were confirmed with a LASSO feature selection framework. For task state, the strongest predictor remained the somatomotor‐to‐basal ganglia connection, but the other task‐based predictors were different. Therefore, our key findings were stable across the statistical framework.

## 4 Discussion

Our data are the first to suggest that, when using the somatomotor network as a seed, the strongest resting‐state connectomic predictor of hand dexterity is connectivity with the basal ganglia in patients with enhancing brain tumors. We further observed that *resting* somatomotor‐to‐basal ganglia connectivity was higher in individuals with *good* dexterity performance, whereas *task‐related* connectivity was higher in individuals with *poor* dexterity performance. Anatomically, participants with poor somatomotor connectivity to the basal ganglia and, consequently, poor dexterity, tended to have lesions involving the gyral white matter underlying the hand knob, suggesting that this region may contain critical connections that enable switching between these networks directly or indirectly. In the subset of better performers, the strongest resting‐state predictor of dexterity was the somatomotor‐to‐salience network with a negative relationship. This finding was consistent with healthy adults from the HCP. Finally, as expected, functional connectivity was a stronger predictor of dexterity than tumor volume or volume of overlap with the descending corticospinal tract.

### 4.1 Somatomotor‐to‐Basal Ganglia Functional Connectivity Predicts Dexterity; However, the Directionality of Its Relationship Inverts From Rest to Task

We observed that *resting* somatomotor‐to‐basal ganglia connectivity was positively related to good performance, whereas *task‐related* connectivity was negatively related to good performance. This suggests that the ability to modulate interactions between these networks or separate common drivers of basal ganglia, salience, and/or somatomotor network connectivity may be related to the ability to perform dexterous movements.

While we are the first to demonstrate the importance of modulating connectomic predictors of function between rest and task in brain tumors, others have shown similar phenomena under different circumstances. (Maddaluno et al. 2024; Vinehout et al. 2022; Greene et al. 2018) For example, the relationship between network modularity/centrality and dexterous performance inverts from rest to task in magnetoencephalography (MEG) data from the HCP. (Maddaluno et al. 2024) Similarly, in Parkinsonism, edgewise connectomic predictors of Stroop task performance change between rest and task and further change directionalities. (Müller‐Oehring et al. 2015) Moreover, comparing rest to task in stroke, connectivity increases between networks necessary for task completion during a task. (Vinehout et al. 2022) (Karim et al. 2020; Marchand et al. 2013)

Therefore, it is possible that attentional demands of a task are related to brain‐behavior relationships. Indeed, attentional performance is related to dynamic functional connectivity (or the ability to flexibly vary connectivity patterns over time). (Fong et al. 2019) Further, previous work has demonstrated that dynamic functional connectivity is impaired in patients with brain tumors. (Moretto et al. 2023) One alternative theory could be that the BOLD signal is inversely related to beta band frequencies but positively related to gamma band frequencies. (Scheeringa et al. 2011) Consistent with this theory, we speculate that a patient with high resting beta synchronization (potentially corresponding to low resting state BOLD synchronization) would have worse task performance due to the tonic inhibitory effects of beta oscillations. (Brittain et al. 2014) Conversely, that same person may have high task‐induced gamma oscillations (potentially associated with high BOLD synchronization) associated with performing difficult tasks. (Doyon et al. 2009; Fischer 2021) Future studies may experimentally examine these phenomena to differentiate potential causes.

Additionally, in our outlier‐excluded data, there was a complete separation of connectivity between individuals able versus unable to complete the dexterity task. Because of the tumor co‐involvement with the hand‐knob gyral white matter in these participants, we suspect that direct involvement of cortico‐basal ganglia fibers disproportionately impacts connectivity and, thereby, dexterity. We suspect that individuals with edematous‐only involvement of this area may alternatively be able to achieve sufficient cortico‐basal ganglia communication through a sufficient number of functional fibers. On the other hand, direct tumor involvement of this path may have more complete disruption, surpassing the brain's intrinsic redundancy.

### 4.2 Somatomotor‐to‐Salience Connectivity Inversely Predicts Dexterity

The finding that the opercular/inferior frontal cortex represents the strongest relationships to dexterity (Figure 1E; Supplementary Materials S6–S7) is not surprising, as it is commonly known to modulate motor control in certain contexts (Gordon et al. 2023); however, the negative direction of the relationship is somewhat less intuitive. It is important to note that network connectivity and phenotypical dysfunction are not directly related, as many networks are normally either inversely or non‐correlated. (Rinne et al. 2018; Newbold et al. 2021; Tomiyama et al. 2022) Further, inhibitory/facilitatory effects cannot be inferred from fMRI. Additionally, increased connectivity may be seen during pathological adaptations, such as in cases of upper extremity disuse that lead to increased functional connectivity between the salience network and the somatomotor network, or in epilepsy. (Newbold et al. 2021) Heightened somatomotor‐to‐salience network connectivity in patients with poor dexterity, therefore, may indicate pathological functional remodeling. (Newbold et al. 2021; Tomiyama et al. 2022)

### 4.3 Implications for Therapeutic Neuromodulation

There is emerging evidence that neuromodulation‐assisted physical therapy may improve recovery in neuro‐oncology patients. (Poologaindran et al. 2022; Chang et al. 2012) In addition to suggesting anatomical circuitry targets, our results have several new implications for treatment paradigms aimed at improving motor control symptoms. Namely, (1) patients with substantial deficits may need different targeting than those with minor deficits (i.e., cortico‐basal ganglia circuits vs. salience‐to‐somatomotor connections, respectively), and (2) stimulation techniques (i.e., inhibitory vs. facilitatory) may need to change depending on whether they are applied during rehabilitation or at rest. Additionally, our data provides empirical support for future studies that will investigate network interactions subserving dexterity in patients with brain tumors. Future studies may also consider applying therapies to modulate cortico‐basal ganglia connectivity in patients who cannot complete dexterous activities through pharmacotherapy, such as levodopa, which has been shown to have some effect in tumor‐related Parkinsonism. (Timpka et al. 2023)

### 4.4 Limitations

Because we analyzed scans that were obtained for clinical purposes, parameters changed during the course of the study due to changes in clinical protocols. We controlled for this through statistical modeling to remove these effects. Additionally, as our sample comprised a heterogeneous group of contrast‐enhancing brain tumors such as glioblastoma, brain metastasis, and radionecrosis, we caution against drawing conclusions regarding direct relationships between specific pathologies and connectivity or dexterity. Other studies have also mixed brain tumors by grade of glioma or primary (glioma) versus metastatic tumors when exploring brain‐behavior relationships. (Otten et al. 2012; Martino et al. 2011; Mallela et al. 2016; Ueda et al. 2022; Yamao et al. 2023) This heterogeneity did not, however, statistically impact our findings.

While studies have looked at brain: ‐behavior relationships in people with brain tumors (for a variety of functions), previous studies have inconsistently controlled for the effects of neurovascular uncoupling. (Otten et al. 2012; Mallela et al. 2016; Yamao et al. 2023; Liouta et al. 2019) Thus, this is a limitation of ours and other studies. Nevertheless, in our study most connectivity edges were not related to tumor overlap of the somatomotor network. Therefore, neurovascular uncoupling alone was unlikely to be the sole contributor to our findings.

As with many studies involving clinical populations, neurovascular uncoupling and persistent noise may impact findings. Nevertheless, our connections of interest were weakly related to framewise displacement, so noise was unlikely a substantial contributor to our results.

### 4.5 Future Studies

Future studies seeking to discover unequivocal causality of the effects of brain tumors on connectivity and, thereby, function, should employ methods less susceptible to neurovascular uncoupling. Such techniques may include cerebral blood flow‐based measures, magnetoencephalography, or measures such as combined electroencephalography plus fMRI or transcranial magnetic stimulation plus fMRI. (Boerger et al. 2023) Additionally, future studies should recruit sufficient samples across grade and/or tumor type to statistically compare these groups. Future studies with larger sample sizes may enable empirical modeling of the relationship between voxelwise lesion location (ideally a voxelwise threshold of 5 participants should be used), tumor type, neurovascular uncoupling connectivity, and functional limitations.

## 5 Conclusion

In conclusion, we discovered the following: (1) The inability to complete dexterous tasks is most associated with *low* somatomotor‐basal ganglia network connectivity during rest but *high* somatomotor‐basal ganglia network connectivity during task; (2) in the subgroup of better dexterous performers, resting somatomotor‐salience connectivity is *higher* in people with *poor* dexterity—a relationship that holds true in healthy adult subjects from the Human Connectome Project, suggesting it has fundamental importance; and (3) connectomic measurements were stronger predictors of dexterous performance than classical variables of tumor (i.e., size, grade, or overlap with the corticospinal tract). These data suggest that connectomic correlates of dexterity are different in resting and task‐based states. Additionally, our data suggest a threshold level of somatomotor‐to‐basal ganglia connectivity is required to accomplish dexterous movements, and, in the cases of appropriately preserved somatomotor‐to‐basal ganglia connectivity, salience‐to‐somatomotor connectivity then becomes the dominant connection facilitating performance in a hierarchical fashion. Hand dexterity appears to be related to complex network interrelationships involving the subcortex, insula, cingulate, and frontal areas. These findings have fundamental implications for both surgical planning and neuromodulation‐based rehabilitation.

## Author Contributions

**Timothy F. Boerger**: conceptualization, data collection, analysis, interpretation, funding, drafting, revising, final approval. **Leon Taquet**: data collection, revising, final approval. **Kaitlin Goetschel**: data collection, revising, final approval. **Sarah Young**: data collection, revising, final approval. **Jennifer Connelly**: revising, final approval. **Jeffrey R. Binder**: revising, final approval. **Brian D. Schmit**: interpretation, revising, final approval. **Max O. Krucoff**: conceptualization and interpretation, project supervision, revising, final approval.

## Funding

This project was funded in part by the Advancing a Healthier Wisconsin Endowment. Data were provided [in part] by the Human Connectome Project, MGH‐USC Consortium (Principal Investigators: Bruce R. Rosen, Arthur W. Toga, and Van Wedeen; U01MH093765) funded by the NIH Blueprint Initiative for Neuroscience Research grant; the National Institutes of Health grant P41EB015896; and the Instrumentation Grants S10RR023043, 1S10RR023401, 1S10RR019307.

## Conflicts of Interest

The authors declare no conflicts of interest.

## Supporting information




**Supplementary Materials**: brb371032‐sup‐0001‐SuppMat.pdf

## Data Availability

Depositing of data into public repositories is not available for this study due to privacy or ethical restrictions. Data will be made available upon reasonable request to the corresponding author.
