# Supplementary material for: Different Brain Network Connectomic Relationships Subserve Hand Dexterity During Task Versus Resting States in People with Brain Tumors
Source: Brain Behav. 2025 Nov 17;15(11):e71032. doi: 10.1002/brb3.71032 (PMC12623447; doi:10.1002/brb3.71032)
Supplement: Supplementary file 1 — Supplementary Materials: brb371032‐sup‐0001‐SuppMat.pdf [file BRB3-15-e71032-s001.pdf]

SUPPLEMENTAL MATERIALS

Supplemental Methods

fMRI Processing

fMRI data were processed with a set of complimentary programs. Advanced Normalization Tools was used to bias correct and register (along with anatomical T1 images) to the Montreal Neurological Institute atlas. FMRIB Software Library supplemented with ICA-AROMA was used to strip the skull from the image, motion correct, smoothing (6mm full-width at half-maximum), subject-level independent component analysis, calculate DVARS. DVARS values >1.5*interquartile range were considered highly motion contaminated and marked for censoring. AFNI was used to detrend, censor motion contaminated volumes with linear interpolation, and bandpass the resulting time series between 0.01 and 0.08 Hz. For task-based connectivity analyses, we used FEAT, without pre-whitening, prior to the lowpass at 0.08 Hz step, to regress out the block design convolved with the hemodynamic response. We extracted network time series data based on the 17-network Yeo 2011(Yeo et al., 2011) atlas supplemented with networks for the basal ganglia, thalamus, and cerebellum. As subcortical structures frequently segregate out as whole networks in ICA studies,(Kalinosky et al., 2017) we elected to keep these structures as distinct networks. Network timeseries were then imported into MATLAB (Natick MA, USA) and FSLNETS was used to calculate partial correlations with Fischer's r to z transformation. Connectivity data were adjusted for age, sex, and lesion volume, and scan parameters using linear regression (fitlm). To examine what parcels of the salience network most strongly predict dexterity, we used the Schaefer 17 network 400 parcel atlas.(Schaefer et al., 2018) We then calculated each salience network parcel's connectivity to each parcel of the somatomotor network and summed the resultants.

Because a few of our participants had brains that were still distorted following standard registration procedures, we serially re-registered these images to the MNI 152 template image. This was particularly a problem in displacing the precentral gyrus posteriorly in patients who had To accomplish this, we used the patient's T1 image registered to the MNI 2mm space as the moving image and the MNI template as the fixed image and re-performed the registration procedure. We then concatenated those transforms with prior transforms and applied them. This was performed up to 7 times because after, 5-7 re-registrations, image alignment had greatly improved in these cases and we began to see diminishing returns. Upon finishing these procedures, we concatenated all transforms and used the concatenated transform to bring the patient's MRI data into standard space.

Because Human Connectome Project fMRI data is preprocessed to remove bias fields, motion, and noise components, we spatially smoothed to 6mm full width at half maximum and bandpass filtered between 0.01 and 0.08 Hz. We then extracted the same network timeseries as our analysis on brain cancer. FSLNETS was likewise used to calculate r to z transformed partial correlations.

In order to balance the covariate adjustments between functional connectivity and hand dexterity, normalized and inverted 9-hole peg test scores were further adjusted for lesion volume. This was performed in MATLAB using the *fitlm* function.

Supplemental Figures

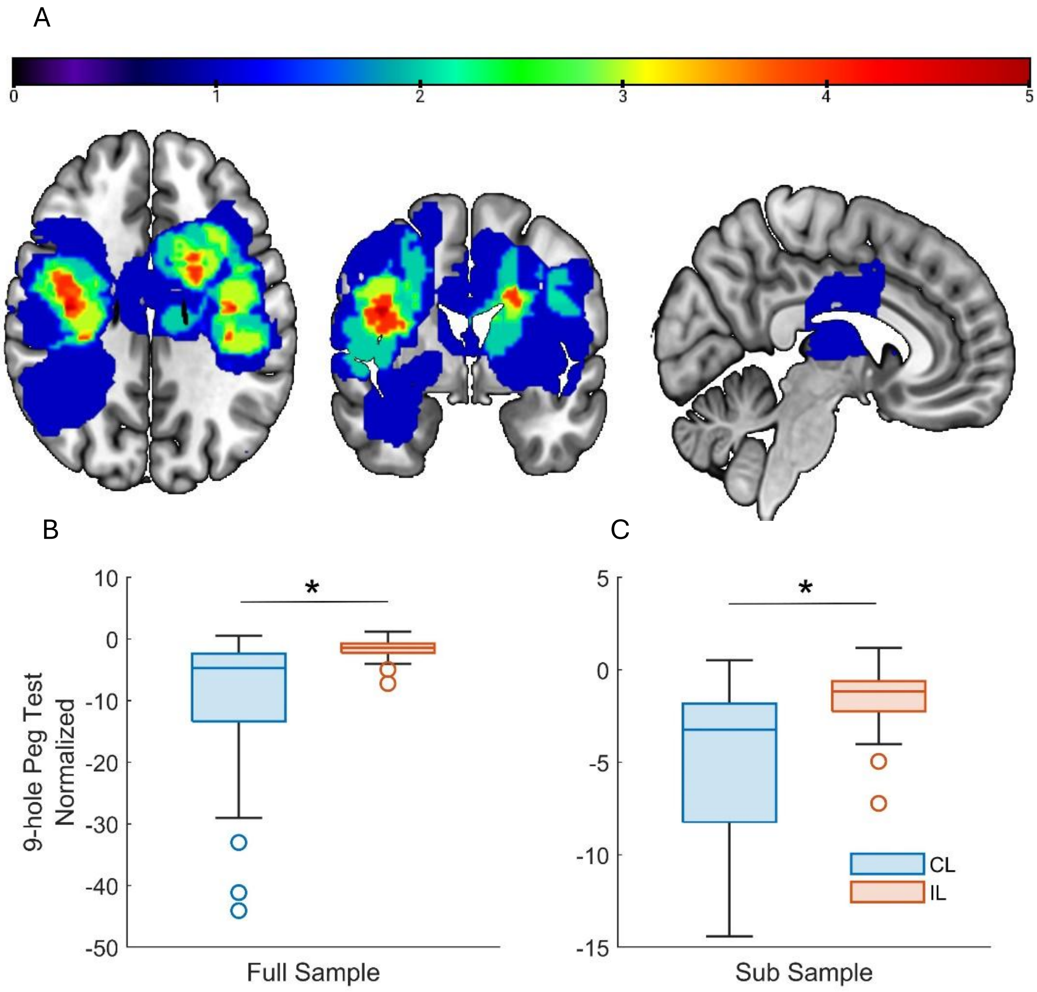

Supplemental Figure 1. Descriptive characteristics of participants. A) Anatomical distribution of brain tumors. Higher values indicate a greater number of tumors in a given area. B) Distribution of 9-hole peg test scores after normalizing based on population norms. C) Distribution of 9-hole peg test data excluding participants unable to complete the task within 120s. *=p<0.05.

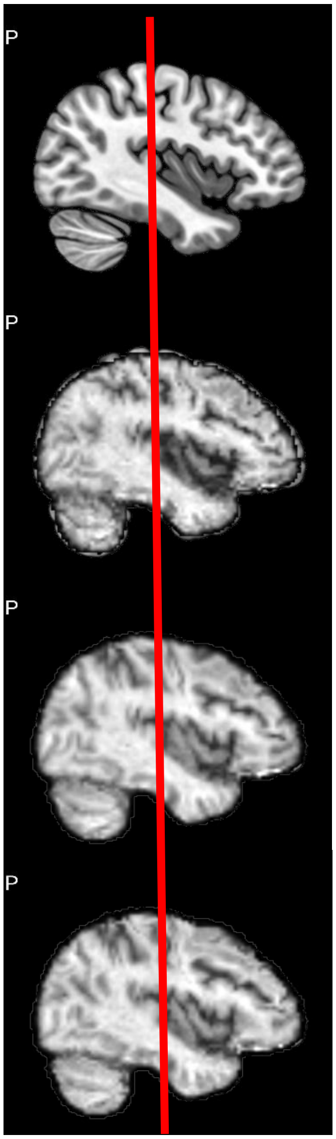

Supplemental Fig 2. Effects of serial registration for spatial normalization of the hand knob and parietal structures. Top to bottom represent MNI standard space, 1-warp, 3-warps, 7-warps respectively. Notice the progressive correction in anterior position of the post-central gyrus and posterior parietal cortex. Vertical red line indicates the position of the central sulcus posterior to the hand knob.

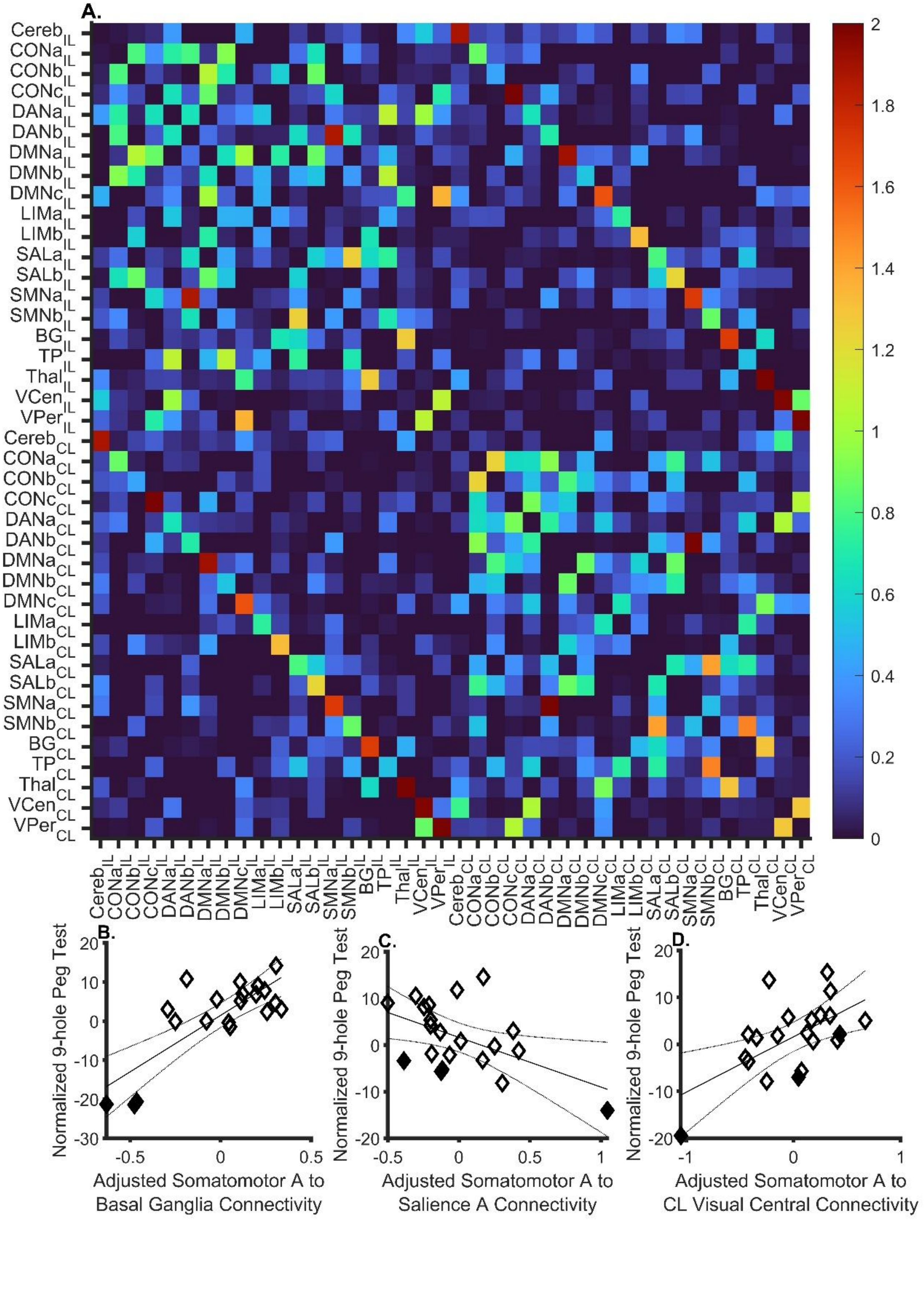
A.
B.
C.
D.
Normalized 9-hole Peg Test
Adjusted Somatomotor A to Basal Ganglia Connectivity
Adjusted Somatomotor A to Salience A Connectivity
Adjusted Somatomotor A to CL Visual Central Connectivity

Supplemental Figure 3. Expanded stepwise regression results for full analysis. A) Average resting state functional connectivity in the full cohort (n=22). B) Partial regression displaying the unique variance in dexterity assumed by the connection from the somatomotor to basal ganglia network connection (β=28.93, $p=4.4*10^{-5}$). C) Partial regression displaying the unique variance in dexterity assumed by the connection from the somatomotor to salience A network connection (β=-10.65, $p=2.6*10^{-2}$). D) Partial regression displaying the unique variance in dexterity assumed by the connection from the somatomotor to contralesional visual central network connection (β=11.79, $p=6.2*10^{-3}$). Regression coefficients differ from in-text values in that the values reported in-text represent the values of each variable at entry, whereas the values here represent regressions after adjusting for the other entered predictors (i.e. fully unique variances assumed). *IL*=ipsilesional. *CL*=contralesional.

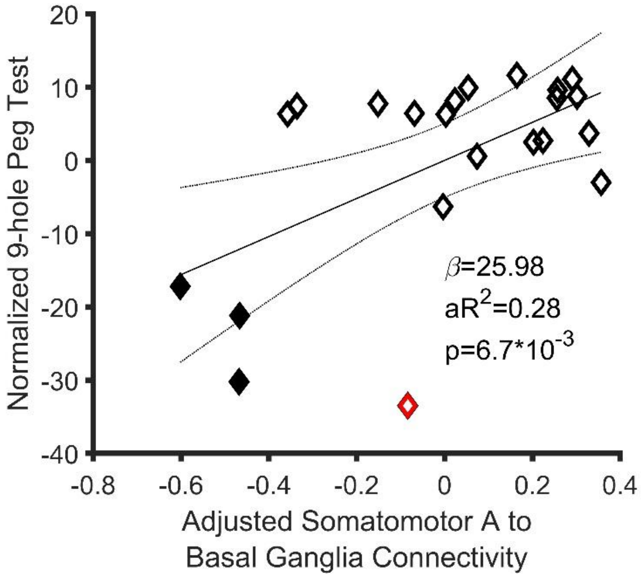

Supplementary Figure 4. Results of stepwise regression predicting dexterity with outlier included. Full sample (n=22) results suggest that the only significant predictor was connectivity from the basal ganglia to the somatomotor network. Outlier is displayed in red.

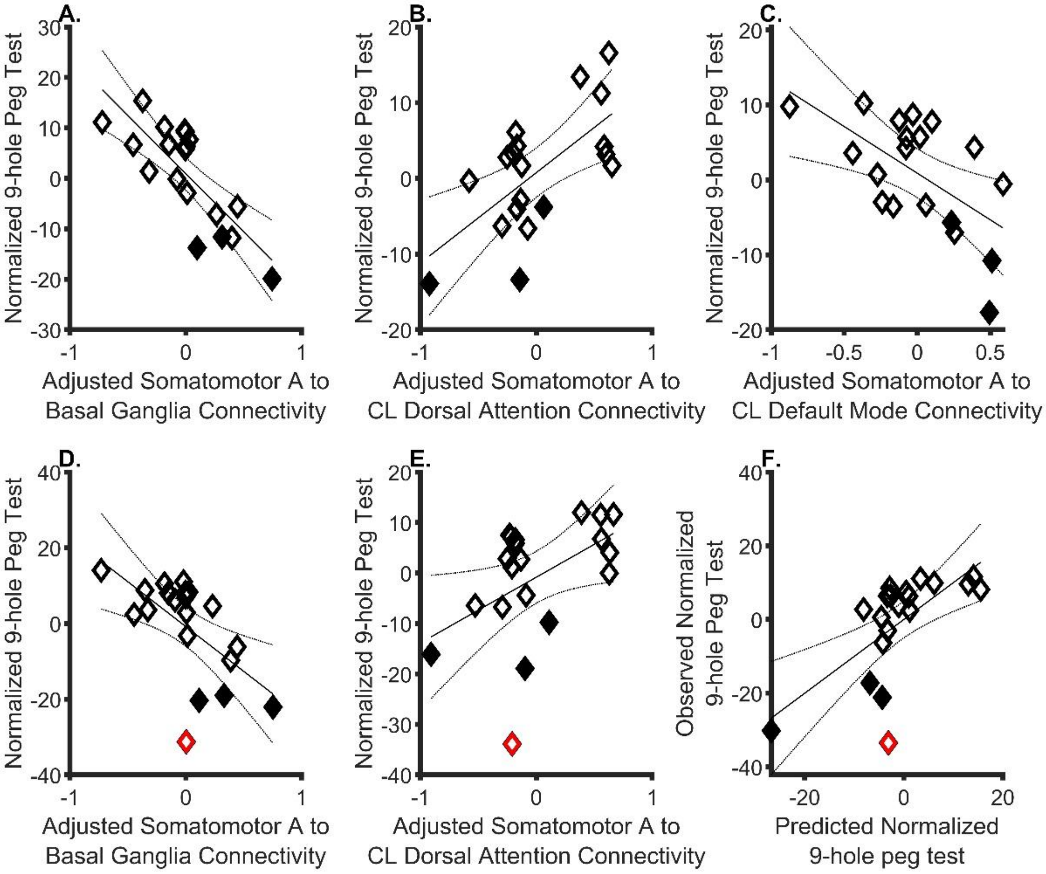

Supplementary Figure 5. Extended data for task-based connectomic prediction of dexterity. A-C) Partial regressions for predictors without 1 outlier (n=19). D-F) Partial and final predicted model with outlier included (n=20). Black diamonds indicate the n=3 participants who were unable to complete the 9-hole peg test. Note that in some pannels, their y-values are higher than those reported in text due to controlling for the variance associated with other variables. Two participants did not have task-based fMRI performed and were excluded from this analysis. A) Prediction of dexterity from basal ganglia to somatomotor connectivity (β= -23.08 (95%CI -32.86 – -13.30), p=0.0001) controlling for contralesional dorsal attention and default mode connectivities. B) Prediction of dexterity by contralesional dorsal attention network connectivity ($F_{4,15}$=13.6, aR^2=0.68, p=0.0002) controlling for basal ganglia and default mode connectivities. C) Prediction of dexterity for contralesional default mode connectivity to the somatomotor network (β= 11.94 (95%CI 4.33 – 19.54), p=0.004) controlling for basal ganglia and dorsal attention connectivity. D) Prediction of dexterity for the basal ganglia to somatomotor network connectivity controlling for contralesional dorsal attention connectivity (β= -23.68 (95%CI -39.49 – -7.86), p=0.006) with outlier (red). E)

Prediction of dexterity from connectivity form the contralesional dorsal attention connectivity to the somatomotor network controlling for the basal ganglia connectivity (β= 13.04 (95%CI 0.84 – 25.23), p=0.037) with outlier (red). F) Predicted dexterity based on basal ganglia and contralesional dorsal attention network connectivity to the somatomotor network vs. observed dexterity with outlier (red) ($F_{3,17}$=6.64, aR^2=0.37, p=0.007). *CL*=contralesional.

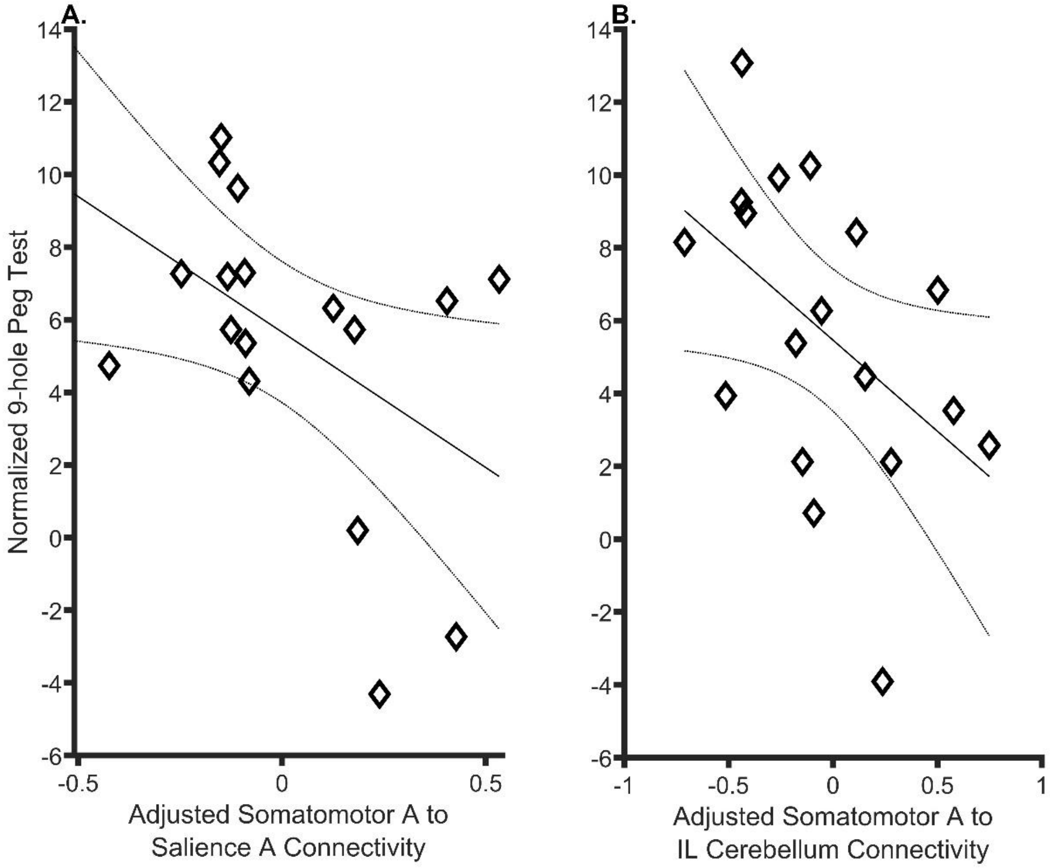

Supplementary Figure 6. Partial regression plots for the subanalysis of patients able to complete the 9-hole peg test (β= -7.46, p=0.038). A) partial regression for the prediction of dexterity from the Salience connectivity to the somatomotor network controlling for ipsilesional cerebellum. B) Partial regression of ipsilesional cerebellum connectivity to

the somatomotor network after controlling for salience connectivity to the somatomotor network (β= -5.00, p=0.049). *IL*=ipsilesional.

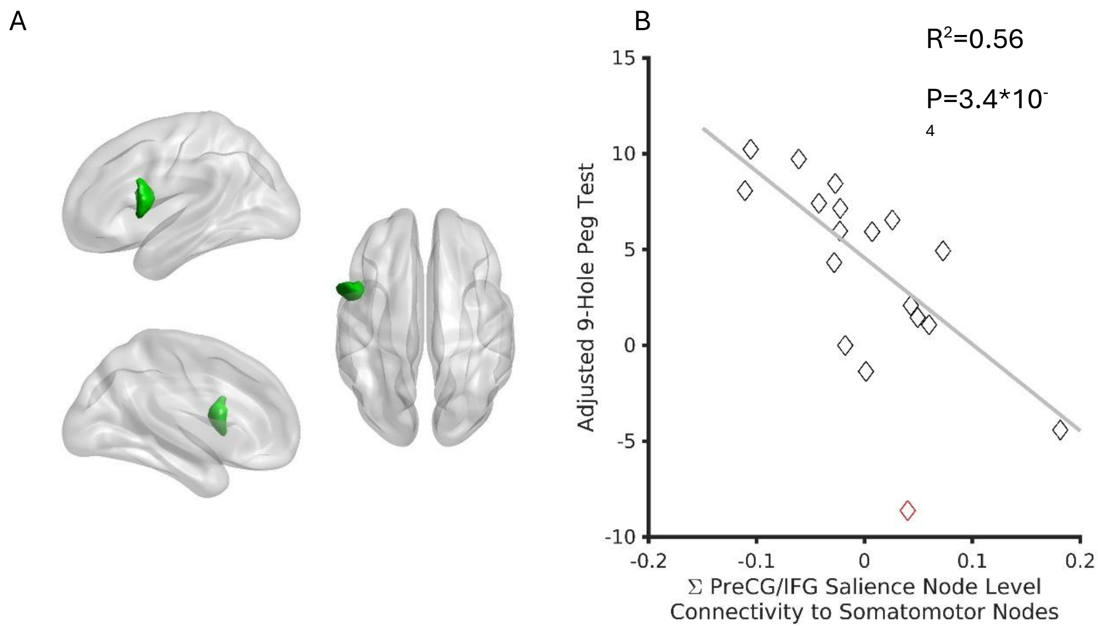

Supplementary Figure 7. Strongest predictor of salience node's sum of connectivity to the somatomotor network. A) Spatial topography of the node that most strongly predicts subsample dexterity. B) regression results of prediction of dexterity. n=1 further outlier is displayed in red. Analyzed sample n=17.

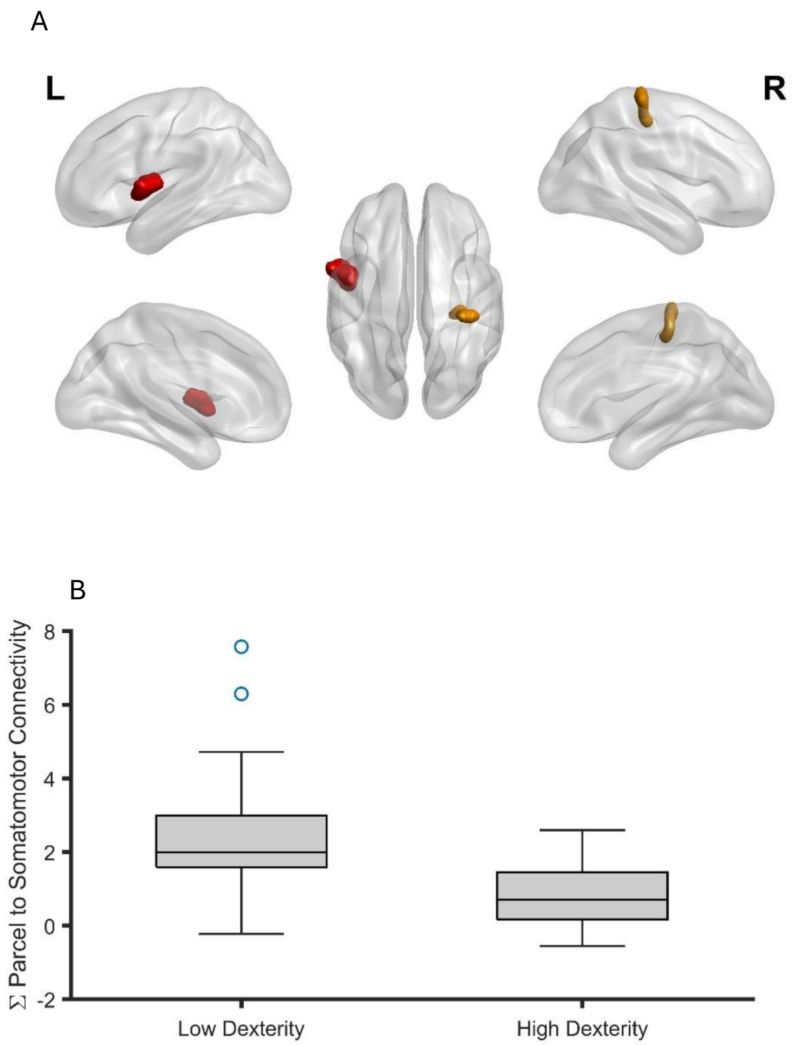

Supplementary Figure 8. Human Connectome Project node level predictors of dexterity. The Salience node of which the sum of connections to the somatomotor network nodes most strongly predicted dexterity was located in the left operculum.

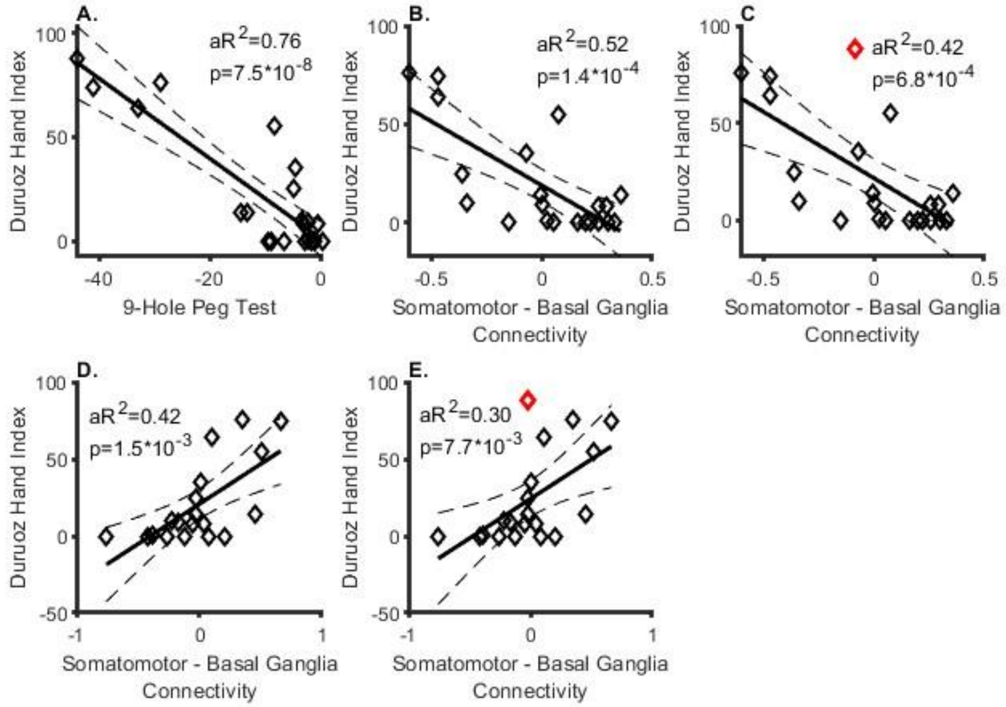

Supplementary Figure 9. Extended data for the interrelationship between 9-hole peg test and Duruoz hand index and connectomic prediction of the Duruoz. A) 9-hole peg test strongly predicts Duruoz hand index. B) Somatomotor connectivity to the basal ganglia significantly predicts Duruoz hand index. C) Prediction of Duruoz hand index scores by somatomotor to basal ganglia connectivity with outlier included. D) Prediction of Duruoz hand index based on task-based somatomotor to basal ganglia connectivity without outlier. E) Prediction of Duruoz hand index based on task-based somatomotor to basal ganglia connectivity with outlier included. For simplicity, connectomic-Duruoz analyses were limited to the somatomotor to basal ganglia connection.

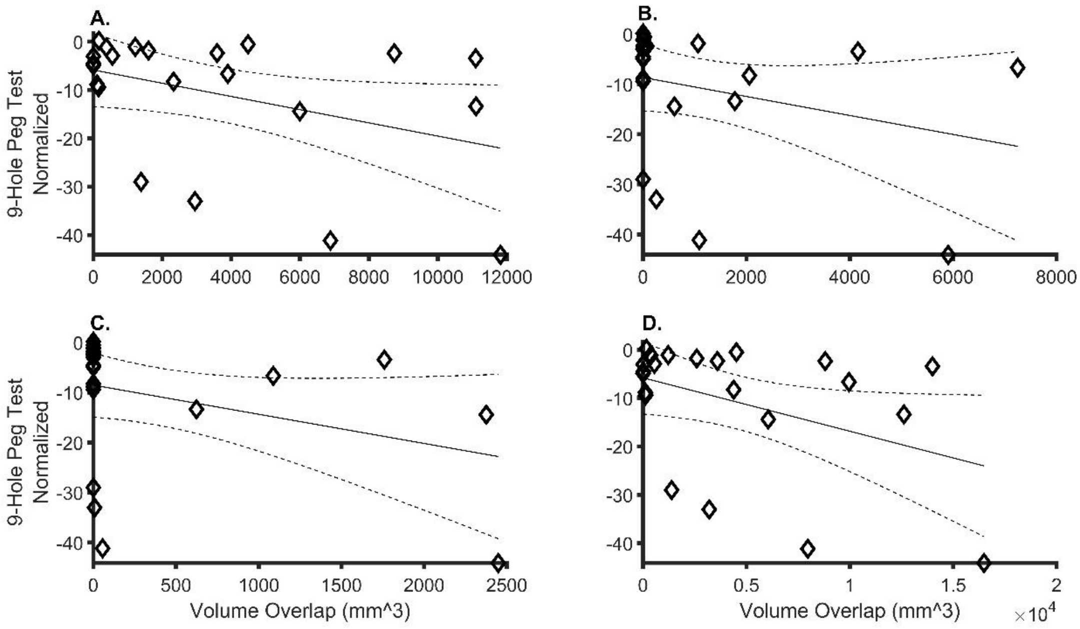

Supplementary Figure 10. Extended data for tumor volume overlap with relevant components of the descending somatomotor system. A) Volume overlap (mm^3) between tumor and corticospinal tract ($F_{2,20}$=3.96, β= -0.001 (95%CI= -0.003 – 0.001), aR^2=0.12, p=0.06). B) volume overlap with the somatomotor network ($F_{2,20}$=2.68, β= -0.006 (95%CI= -0.031 – 0.002), aR^2=0.07, p=0.12). C) Overlap with specifically the hand knob component of the somatomotor network ($F_{2,20}$=1.83, β= -0.002 (95%CI= -0.005 – 0.001), aR^2=0.04, p=0.19). D) Overlap with the whole somatomotor network and corticospinal tract combined ($F_{2,20}$=4.2, β= -0.001 (95%CI= -0.002 – 0.000), aR^2=0.13, p=0.054).

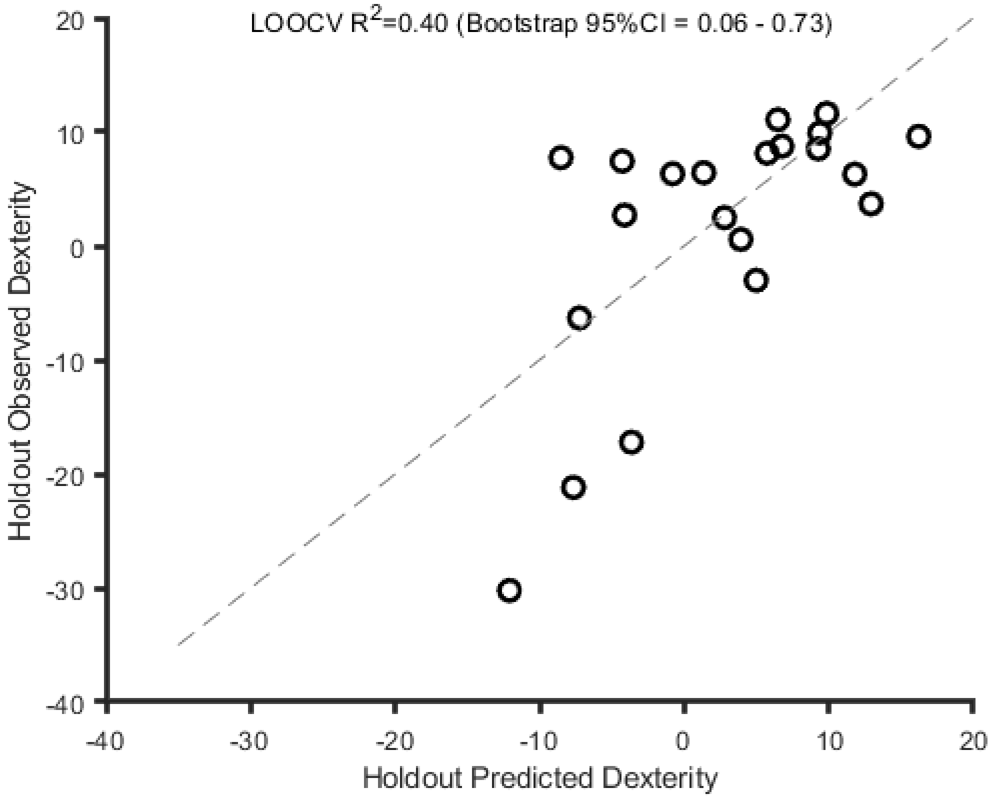

Supplementary Figure 11. Predicted contralesional hand dexterity based on stepwise linear regression. LASSO feature selection (supplementary figure 12) resulted in identical features. Therefore, Ordinary Least Squares regression using on LASSO regularized feature selection results in the same data as this figure. Data are held out data (leave one out cross-validation) vs observed dexterity. Dexterity data were normalized based on published normative data stratified by age, sex, and hand and adjusted based on covariates of tumor volume. Dashed line represents a slope of 1. Inset text presents R2 statistic along with the 95% confidence interval based on bootstrap (100,000 resamples).

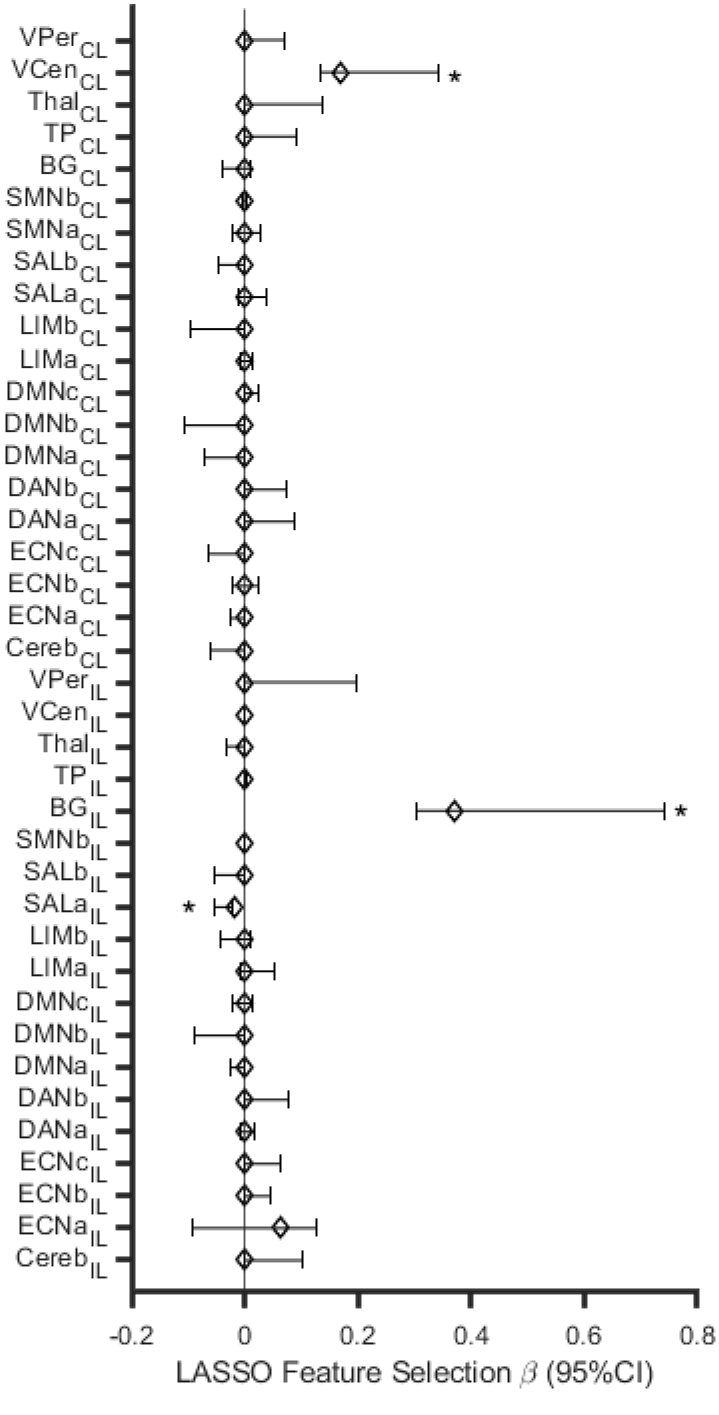

VPer_CL
VCen_CL
Thal_CL
TP_CL
BG_CL
SMNb_CL
SMNa_CL
SALb_CL
SALa_CL
LIMb_CL
LIMa_CL
DMNc_CL
DMNb_CL
DMNa_CL
DANb_CL
DANa_CL
ECNc_CL
ECNb_CL
ECNa_CL
Cereb_CL
VPer_IL
VCen_IL
Thal_IL
TP_IL
BG_IL
SMNb_IL
SALb_IL
SALa_IL
LIMb_IL
LIMa_IL
DMNc_IL
DMNb_IL
DMNa_IL
DANb_IL
DANa_IL
ECNc_IL
ECNb_IL
ECNa_IL
Cereb_IL
*
*
*
-0.2
0
0.2
0.4
0.6
0.8
LASSO Feature Selection β (95%CI)

Supplementary Figure 12. Predictor of 9HPT using a cross-validated LASSO regression model. Connectivity data from n=21 participants were entered into a LASSO regression with leave-one out cross-validation and bootstrap (1,000,000 re-samples) in R. Bayesian optimization was used to identify the optimal lambda parameter. Data are LASSO regularized coefficients (betas) along with bootstrap 95% confidence intervals. Asterisks denote features which have 95% confidence intervals do not cross 0. These data are stage 1 of the 2-stage LASSO feature selection and Ordinary Least Squares regression. Stage 2 of the LASSO feature selection and OLS regression (i.e. multi-linear OLS) are identical to Supplementary Figure 11. *Cereb=cerebellum, ECN=executive control network, DAN=dorsal attention network, DMN=default mode network, LIM=limbic network, SAL=salience/ventral attention network, SMN=somatomotor network, BG=basal ganglia, TP=temporalparietal network, Thal=thalamus, VCen=visual central network, VPer=visual peripheral network. a/b/c = respective subnetworks a/b/c. IL=ipsilesional, CL=contralesional.*

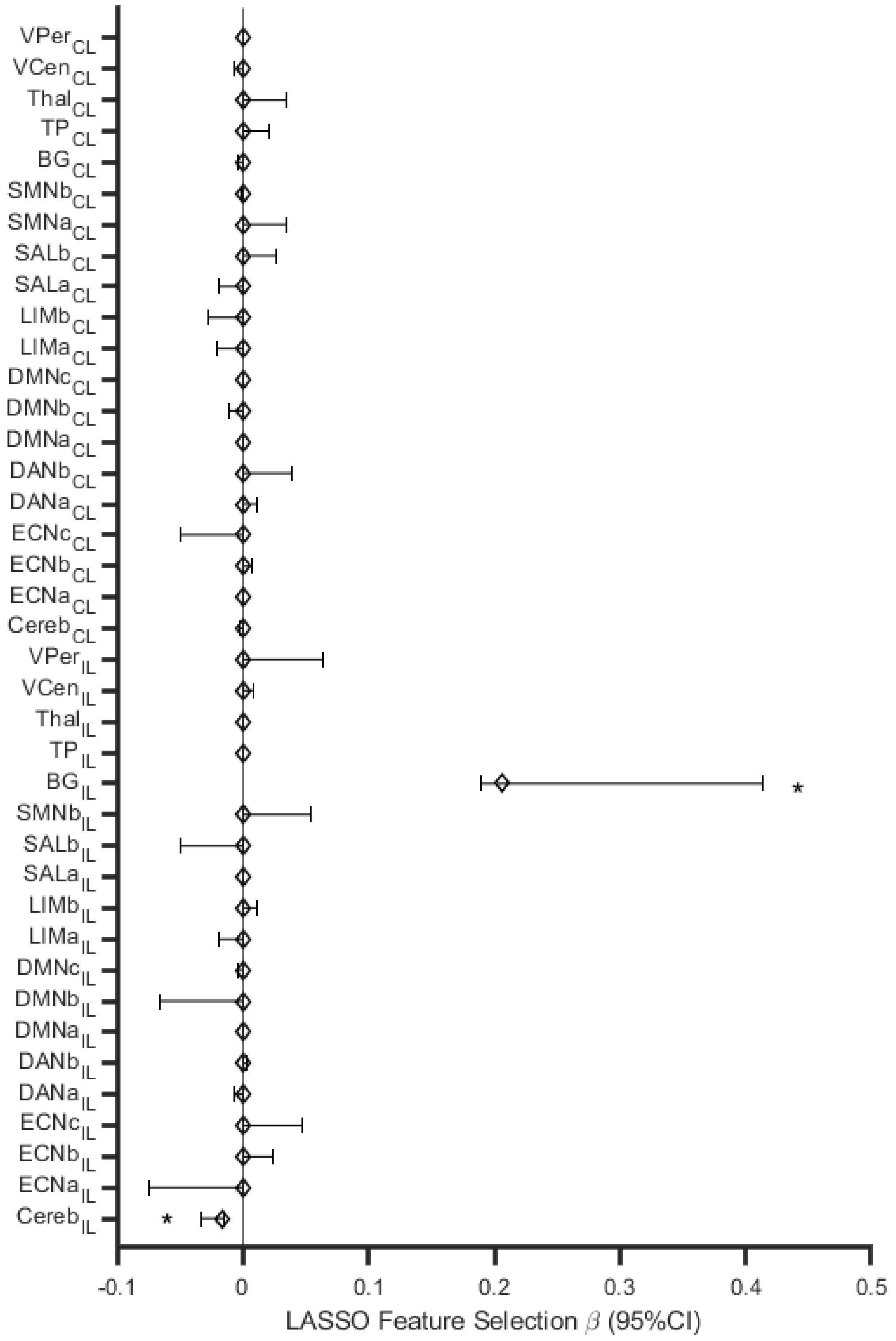

VPer_CL
VCen_CL
Thal_CL
TP_CL
BG_CL
SMNb_CL
SMNa_CL
SALb_CL
SALa_CL
LIMb_CL
LIMa_CL
DMNc_CL
DMNb_CL
DMNa_CL
DANb_CL
DANa_CL
ECNc_CL
ECNb_CL
ECNa_CL
Cereb_CL
VPer_IL
VCen_IL
Thal_IL
TP_IL
BG_IL
SMNb_IL
SALb_IL
SALa_IL
LIMb_IL
LIMa_IL
DMNc_IL
DMNb_IL
DMNa_IL
DANb_IL
DANa_IL
ECNc_IL
ECNb_IL
ECNa_IL
Cereb_IL
*
*
-0.1
0
0.1
0.2
0.3
0.4
0.5
LASSO Feature Selection β (95%CI)

Supplementary Figure 13. Results of LASSO feature selection with Leave-One Out Cross Validation (LOOCV) and Bootstrap for dataset including n=1 outlier. Data represent LASSO regularized coefficients along with bootstrapped 95% confidence intervals based on 1,000,000 bootstrap re-samples. Asterisks denote features which have 95% confidence intervals do not cross 0. With n=1 outlier included, ipsilesional somatomotor to basal ganglia connectivity remained the strongest predictor.

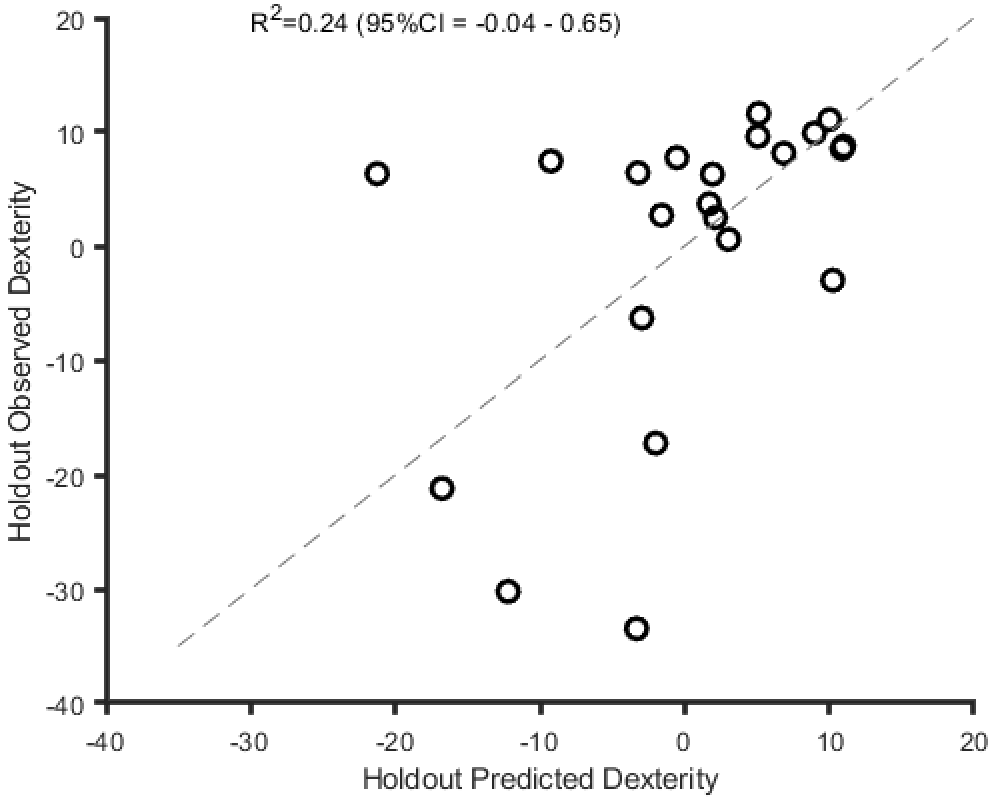

Supplementary Figure 14. Results of OLS regression with LOOCV for analysis with n=1 outlier. Goodness of fit is substantially reduced with this outlier compared to outlier-removed data. Inset text presents R^2 along with the 95% confidence interval based on bootstrap (100,000 resamples).

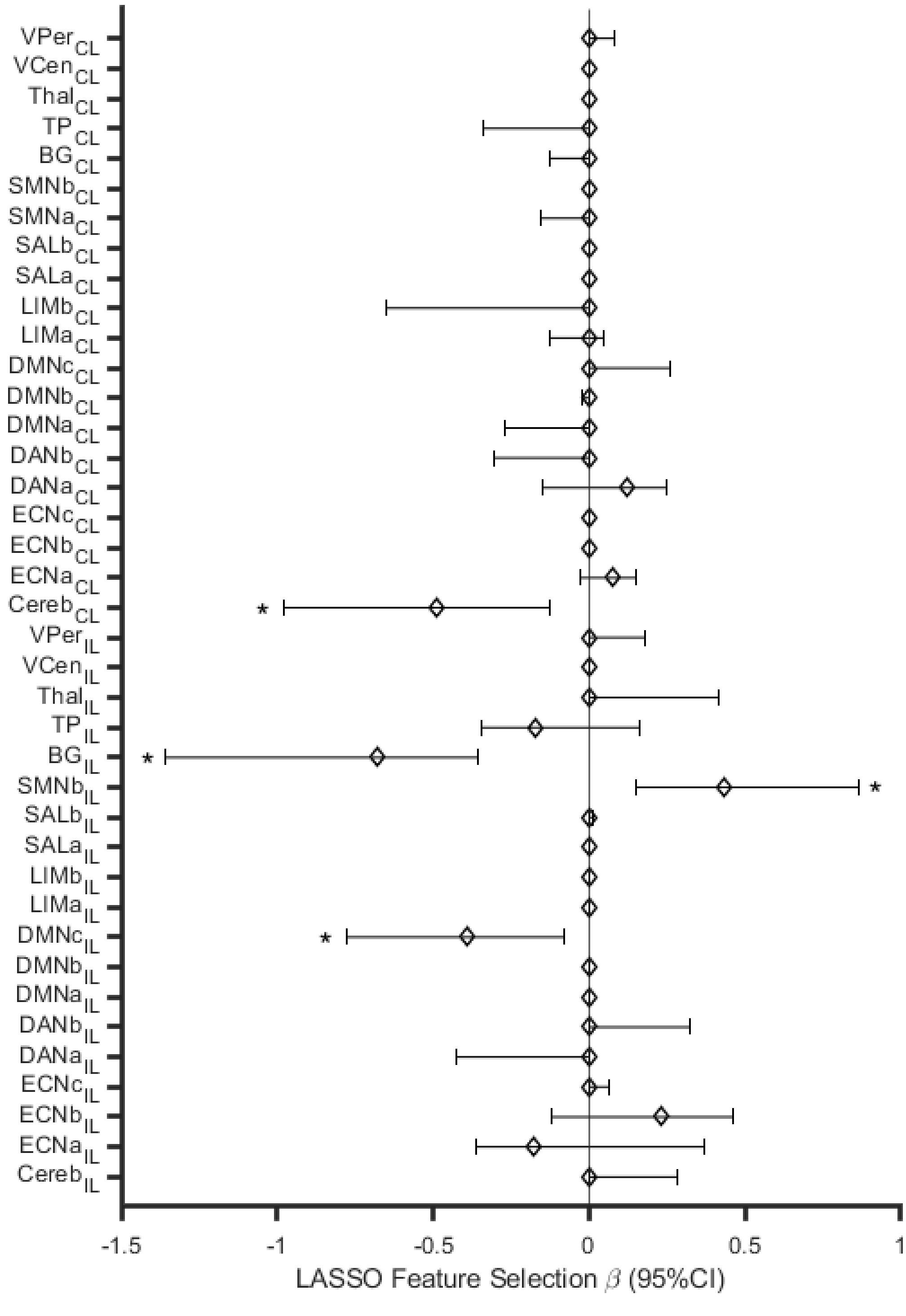

VPer CL
VCen CL
Thal CL
TP CL
BG CL
SMNb CL
SMNa CL
SALb CL
SALa CL
LIMb CL
LIMa CL
DMNc CL
DMNb CL
DMNa CL
DANb CL
DANa CL
ECNc CL
ECNb CL
ECNa CL
Cereb CL
VPer IL
VCen IL
Thal IL
TP IL
BG IL
SMNb IL
SALb IL
SALa IL
LIMb IL
LIMa IL
DMNc IL
DMNb IL
DMNa IL
DANb IL
DANa IL
ECNc IL
ECNb IL
ECNa IL
Cereb IL
*
*
*
*
-1.5
-1
-0.5
0
0.5
1
LASSO Feature Selection β (95%CI)

Supplementary Figure 15. Cross validated and bootstrapped feature selection based on LASSO regression. Connectivity was from the somatomotor to networks listed on y-axis. Data are LASSO regression coefficients along with bootstrap (1,000,000 resamples) 95% confidence intervals. Asterisks denote features which have 95% confidence intervals do not cross 0. Somatomotor to basal ganglia remained the strongest predictor of dexterity and, like the stepwise regression results, was inversely related to dexterity scores.

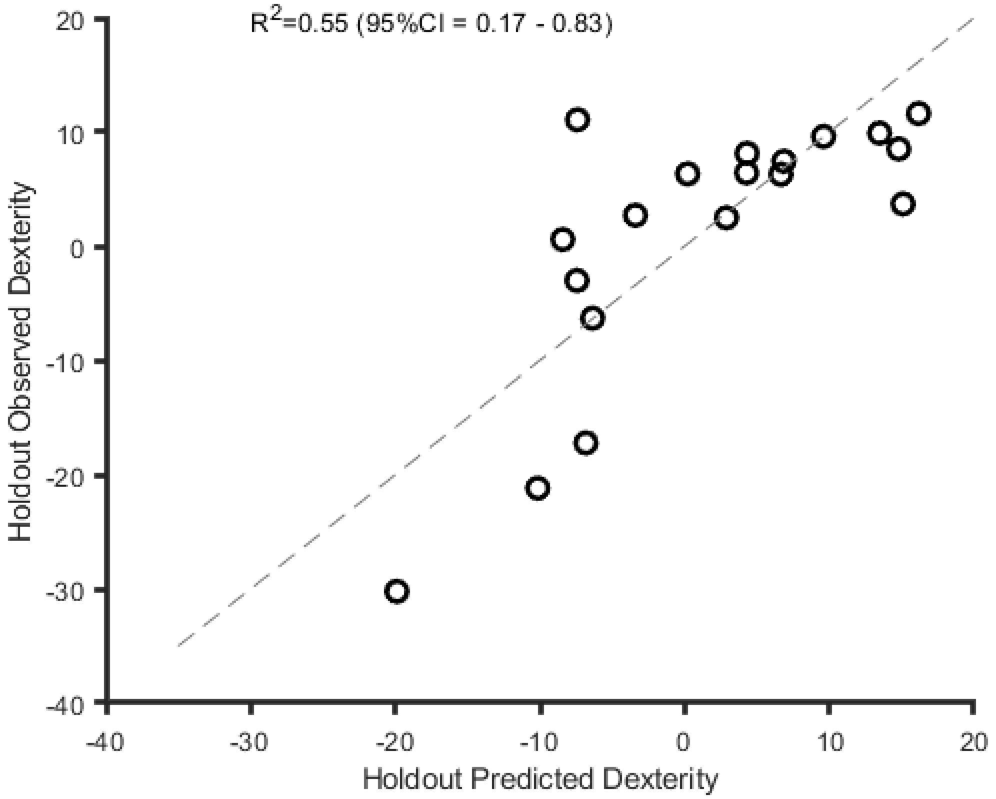

Supplementary Figure 16. Predictive accuracy of task-based functional connectivity on contralesional hand dexterity based on LASSO feature selection. Selected features were entered into OLS regression with LOOCV. LOOCV predicted scores are plotted against observed scores. R^2 and bootstrap 95% confidence interval are provided in the text inset.

References

Kalinosky, B. T., Berrios Barillas, R., & Schmit, B. D. (2017). Structurofunctional resting-state networks correlate with motor function in chronic stroke. *Neuroimage Clin*, *16*, 610-623. https://doi.org/10.1016/j.nicl.2017.07.002

Schaefer, A., Kong, R., Gordon, E., Laumann, T., Zuo, X., Holmes, A.,...Yeo, B. (2018). Local-Global Parcellation of the Human Cerebral Cortex from Intrinsic Functional Connectivity MRI. *Cerebral cortex (New York, N.Y. : 1991)*, *28*(9). https://doi.org/10.1093/cercor/bhx179

Yeo, B., Krienen, F., Sepulcre, J., Sabuncu, M., Lashkari, D., Hollinshead, M.,...Buckner, R. (2011). The organization of the human cerebral cortex estimated by intrinsic functional connectivity. *Journal of Neurophysiology*, *106*(3). https://doi.org/10.1152/jn.00338.2011
